# The acute effect of glucagon on components of energy balance and glucose homoeostasis in adults without diabetes: a systematic review and meta-analysis

**DOI:** 10.1038/s41366-022-01223-y

**Published:** 2022-09-19

**Authors:** James Frampton, Chioma Izzi-Engbeaya, Victoria Salem, Kevin G. Murphy, Tricia M. Tan, Edward S. Chambers

**Affiliations:** 1 Section for Nutrition Research, Department of Metabolism, Digestion and Reproduction, Faculty of Medicine, Imperial College London, London, W12 0NN UK; 2 Section of Endocrinology and Investigative Medicine, Department of Metabolism, Digestion and Reproduction, Faculty of Medicine, Imperial College London, London, W12 0NN UK; 3 Department of Bioengineering, Faculty of Engineering, Imperial College London, London, SW7 2BX UK

**Keywords:** Obesity, Obesity

## Abstract

**Objective:**

Using a systematic review and meta-analysis, we aimed to estimate the mean effect of acute glucagon administration on components of energy balance and glucose homoeostasis in adults without diabetes.

**Methods:**

CENTRAL, CINAHL, Embase, MEDLINE, PubMed, and Scopus databases were searched from inception to May 2021. To be included, papers had to be a randomised, crossover, single- or double-blind study, measuring *ad libitum* meal energy intake, energy expenditure, subjective appetite, glucose, and/or insulin following acute administration of glucagon and an appropriate comparator in adults without diabetes. Risk of bias was assessed using the Revised Cochrane Risk of Bias Tool for Randomized trials with additional considerations for cross-over trials. Certainty of evidence was assessed using the GRADE approach. Random-effect meta-analyses were performed for outcomes with at least five studies. This study is registered on PROSPERO (CRD42021269623).

**Results:**

In total, 13 papers (15 studies) were considered eligible: energy intake (5 studies, 77 participants); energy expenditure (5 studies, 59 participants); subjective appetite (3 studies, 39 participants); glucose (13 studies, 159 participants); insulin (12 studies, 147 participants). All studies had some concerns with regards to risk of bias. Mean intervention effect of acute glucagon administration on energy intake was small (standardised mean difference [SMD]: –0.19; 95% CI, –0.59 to 0.21; *P* = 0.345). Mean intervention effect of acute glucagon administration on energy expenditure (SMD: 0.72; 95% CI, 0.37–1.08; *P* < 0.001), glucose (SMD: 1.11; 95% CI, 0.60–1.62; *P* < 0.001), and insulin (SMD: 1.33; 95% CI, 0.88–1.77; *P* < 0.001) was moderate to large.

**Conclusions:**

Acute glucagon administration produces substantial increases in energy expenditure, and in circulating insulin and glucose concentrations. However, the effect of acute glucagon administration on energy intake is unclear. Insufficient evidence was available to evaluate the acute effect of glucagon on subjective appetite.

## Introduction

Obesity is a global health burden associated with increased cardiometabolic disease risk and mortality [1, 2]. Lifestyle modification centred on dietary restriction and increased physical activity is the first-line treatment. However, adherence to such programmes is typically poor and weight-loss often modest [3, 4]. Bariatric surgery produces substantial and sustained weight loss [5], but it is not universally available or acceptable to all eligible patients. Therefore, pharmacological interventions are urgently required for long-term weight-loss.

Glucagon is a 29 amino acid polypeptide synthesised by the alpha cells of the pancreatic islets, which acts via the glucagon receptor (GCGR) to exert various physiological effects [6]. Glucagon is primarily known for its role in glucose homoeostasis [7], but has also been identified as a key regulator of amino acid metabolism [8]. Furthermore, glucagon is implicated in the stress response, being released under conditions of psychological and metabolic stress [9], including prolonged fasting [10] and acute exercise [11]. Evidence from rodent models have demonstrated that glucagon can also regulate energy balance, acting to concurrently raise energy expenditure [12] and suppress energy intake [13], and thus influence body weight [14]. This is thought to be achieved via both direct mechanisms, including GCGR activation on target tissues such as the hypothalamus [15] and brown adipose tissue [16], and indirect mechanisms, including the release of other hormones such as fibroblast growth factor 21 [17] and catecholamines [18].

GCGR agonism has consequently been identified as a possible therapeutic target for obesity, and a number of studies have investigated the effects of acute glucagon administration on energy intake and energy expenditure in humans. However, the magnitude and/or direction of effects following glucagon administration has been mixed for these components [19–23], likely attributable to differences in study design. Indeed, effects attributed to glucagon are frequently confounded by co-infusion of other bioactive peptides, such as somatostatin [24, 25]. Moreover, many studies do not include an appropriate control arm, instead favouring pre-post designs that do not exclude the effect of time on observed responses [26–28].

We therefore conducted a systematic review and random-effects meta-analysis to estimate the mean effects of acute glucagon administration on energy intake, energy expenditure, and subjective hunger in adults without diabetes. Furthermore, mean glucose and insulin responses following acute glucagon administration were also estimated due to their influence on energy balance [29, 30] and recognised association with glucagon.

## Methods

### Registration

This Review was registered in the international prospective register of systematic reviews (PROSPERO; registration number: CRD42021269623) and written in accordance with the recommendations outlined in the PRISMA 2020 statement [31].

### Eligibility criteria

#### Population

We included randomised, controlled, single- or double blind, crossover studies in adults (>18 years old) of any body mass index (BMI) value. Studies performed in current smokers, pregnant individuals, or individuals with a history of chronic disease (including type 1 and type 2 diabetes) were excluded.

#### Intervention

Administration of glucagon via any route (intravenous, intramuscular, intranasal) for less than 24 h while at rest. Studies which administered glucagon for longer than 24 hours or co-infused pharmacological agents (e.g. somatostatin) were excluded. Studies could be performed in the fasted or postprandial state.

#### Comparator

To be included, studies must have performed a time-and energy matched control arm that administered an energy-free control agent (e.g. saline) in place of glucagon.

#### Outcome

Studies measuring energy intake, energy expenditure, subjective appetite, glucose, and/or insulin were included.

We only considered studies written in the English language and published in peer-reviewed journals. Conference abstracts were excluded. If methodology and/or participant characteristics were not described sufficiently to determine study eligibility, corresponding authors were contacted. If the author did not respond, or could not provide the required information, the study was excluded.

### Information sources and search strategy

J.F. searched CENTRAL, CINAHL, Embase, MEDLINE, PubMed, and Scopus databases on 24 May 2021. Embase and Medline databases were accessed via Ovid, and the CINAHL database was accessed via EBSCOhost. All databases were searched from inception to 24 May 2021.

The search strategy was developed based on the PICO format, with additional concepts incorporated to exclude pre-clinical studies. Full details of the search strategy are provided in Supplementary Appendix A. No limits were used during any database search.

Backward (using Google Scholar) and forward citation searching of eligible papers was also performed by J.F on 23 July 2021.

### Selection process

Results of each database search were imported into Covidence systematic review software (Veritas Health Innovation, Australia). Duplicate results were automatically detected and removed by Covidence. Title and abstracts were then independently screened by JF and ESC, with each paper being classified as ‘yes’,’no’ or ‘maybe’. Papers classified as ‘yes’ or ‘maybe’ by both JF and ESC continued to the full-text screening phase. All disputes (papers with a ‘yes’ or ‘maybe’ *and* a ‘no’ vote) were resolved prior to conducting full-text screening. Full texts of each paper were then accessed and independently classified as ‘yes’ or ‘no’ by JF and ESC. Papers classified as ‘yes’ continued to the data extraction phase. Disputes following full-text screening (papers with a ‘yes’ *and* a ‘no’ vote) were resolved via a meeting with all authors prior to data extraction.

### Data collection

Corresponding authors for all eligible studies were first contacted for raw study data. If authors did not respond or could not provide raw study data, data were extracted from the published manuscript. WebPlotDigitizer Version 4.2 (Ankit Rohatgi, USA) was used to extract data from papers that only presented data in a figure.

When data were displayed inadequately (e.g. clustering of data points, overlapping of error bars) or data were not reported in published manuscript or supplementary materials (despite methods stating measurements had been taken), the paper was no longer considered eligible and excluded from analysis.

Data were collected by a single author (JF) and stored in an electronic spreadsheet (Excel 2016, Microsoft Corporation, USA). If data were presented from multiple glucagon doses, only data from the highest dose was collected. ESC checked the accuracy of collected data by comparing the results stored in the electronic spreadsheet with those in the published manuscript or raw study data.

### Data items

#### Eligible outcomes were defined as follows

Energy intake—total ad libitum energy intake at the first meal presented to participants following the administration of glucagon and comparator. Measured in kcal, kJ, or grams.

Energy expenditure—change in energy expenditure (pre- vs post-administration) in the glucagon and comparator arms, in which pre-administration is a baseline measurement prior to glucagon/comparator administration and post-administration is a measurement at least 30 min after initial glucagon/comparator administration (to allow sufficient time for an effect to be observed). Alternatively, baseline and at least two other timepoints during the glucagon and comparator arms, or total energy expenditure during the glucagon and comparator arms. Measured in kcal, kJ, or V̇O_2_.

Subjective appetite—assessed at baseline and at least two other timepoints during the glucagon and comparator arms. Alternatively, total or incremental area under the curve (AUC) for the glucagon and comparator arms. Measured by a visual analogue scale (VAS) or other questionnaire assessing a domain relating to appetite (e.g. hunger, pleasantness, prospective consumption, fullness) or a composite appetite score.

Glucose—assessed at baseline and at least two other timepoints during the glucagon and comparator arms. Alternatively, total or incremental AUC for the glucagon and comparator arms. Measured in serum or plasma.

Insulin—assessed at baseline and at least two other timepoints during the glucagon and comparator arms. Alternatively, total or incremental AUC for the glucagon and comparator arms. Measured in serum or plasma.

Pre-vs post administration values were accepted for energy expenditure (but not subjective appetite, glucose, or insulin) due to energy expenditure measurements being performed over extended time periods (e.g. 10–30 min) compared to measurements taken at single time points.

The following data items were also collected relating to paper, participant, and intervention characteristics: author(s), year of publication, sample size, proportion of males, participant age, participant BMI, degree of blinding, route of administration, glucagon dose, and duration of administration.

### Risk of bias assessment

Risk of bias assessment was performed by a single author (JF). Risk of bias of included studies was assessed using the Revised Cochrane Risk of Bias Tool for Randomized trials (RoB 2.0) with additional considerations for cross-over trials. Risk of bias was assessed using the following domains: bias arising from the randomization process; bias arising from period and carryover effects; bias due to deviations from intended intervention; bias due to missing outcome data; bias in the measurement of the outcome; and bias in the selection of the reported result. Risk of bias assessment was performed for each outcome (energy intake, energy expenditure, subjective appetite, glucose, insulin), in which the risk of bias of each individual study was determined by the highest risk of bias level attained in any of the assessed domains. Studies were not excluded based on the risk of bias assessment.

### Data synthesis

Data were collated and grouped by outcome (energy intake, energy expenditure, subjective appetite, glucose, insulin). Standard errors and 95% confidence intervals (CIs) were converted to standard deviations. For energy expenditure, subjective appetite, glucose, and insulin outcomes only, and for studies in which only time-series data were reported, total AUC was calculated for glucagon and comparator arms using the maximum number of timepoints available. If data were extracted from figures using WebPlotDigitizer, standard deviations of AUCs were estimated using the AUC of values depicted by the corresponding top or bottom error bars. If multiple data types were presented (AUC, time points, and/or pre- vs post-administration), the order of priority for extraction was as follows: AUC > time points > pre- vs post-administration.

Standardised mean differences (SMDs) were then calculated for each study as described by Higgins et al. [2]. When raw study data were not available, a correlation coefficient of 0.5 was assumed to calculate the standard error of the SMD [27]. Sensitivity analyses using correlation coefficients of 0.3, 0.7, and 0.9 were performed to assess the robustness of findings to this assumption.

A random-effects meta-analysis model was selected as the effect of glucagon administration on outcomes was expected to vary across studies due to differences in participant and intervention characteristics. This model assumes a distribution of true effect sizes across studies and provides an estimate of the mean intervention effect of this distribution [32, 33]. Between-study variance (τ^2^) was estimated using the Hartung-Knapp-Sidik-Jonkman method [34, 35], with summary effect CIs being calculated using the Wald-type method [36]. Results of syntheses were presented using forest plots. Leave-one-out meta-analysis was also performed to identify studies that have a large influence on the summary effect estimate.

Statistical heterogeneity was assessed using the τ^2^ and I^2^ statistics alongside their corresponding 95% CIs. Random-effects 95% prediction intervals (PIs) were also calculated to facilitate the interpretation of statistical heterogeneity by providing an expected range of intervention effects in similar future studies. Mixed-effects meta-regression using the Knapp and Hartung adjustment [37] was used to explore possible causes of statistical heterogeneity for outcomes with at least ten studies. Separate univariable models were created for each potential moderator: (i) route of administration, and (ii) total glucagon dose. The choice of moderators was made post-hoc.

Potential publication bias was assessed via visual inspection of contour-enhanced funnel plots [38] and statistically by Egger’s regression test for outcomes containing at least 10 studies. Trim and fill analyses (L_0_ estimator) were used when publication bias was suspected to explore its impact on effect sizes [39].

All analyses were performed in R version 4.1.0 [40] using the R package ‘metafor’ [41] by one author (JF). Random-effects meta-analysis was only performed for outcomes with at least five studies [42]. Individual study effects were described for outcomes with less than five studies.

### Certainty of evidence assessment

Certainty of evidence was assessed by two authors (JF and ESC) using the GRADE approach [43, 44]. Certainty of evidence was assessed using the following domains: study limitations, consistency of effect, imprecision, indirectness, and publication bias. Estimated effect of each outcome was independently classified as high (true effect is similar to the estimated effect), moderate (true effect is probably close to the estimated effect), low (true effect might be markedly different from the estimated effect), or very low (true effect is probably markedly different from the estimated effect) certainty of evidence.

## Results

### Study selection

**Fig. 1:**
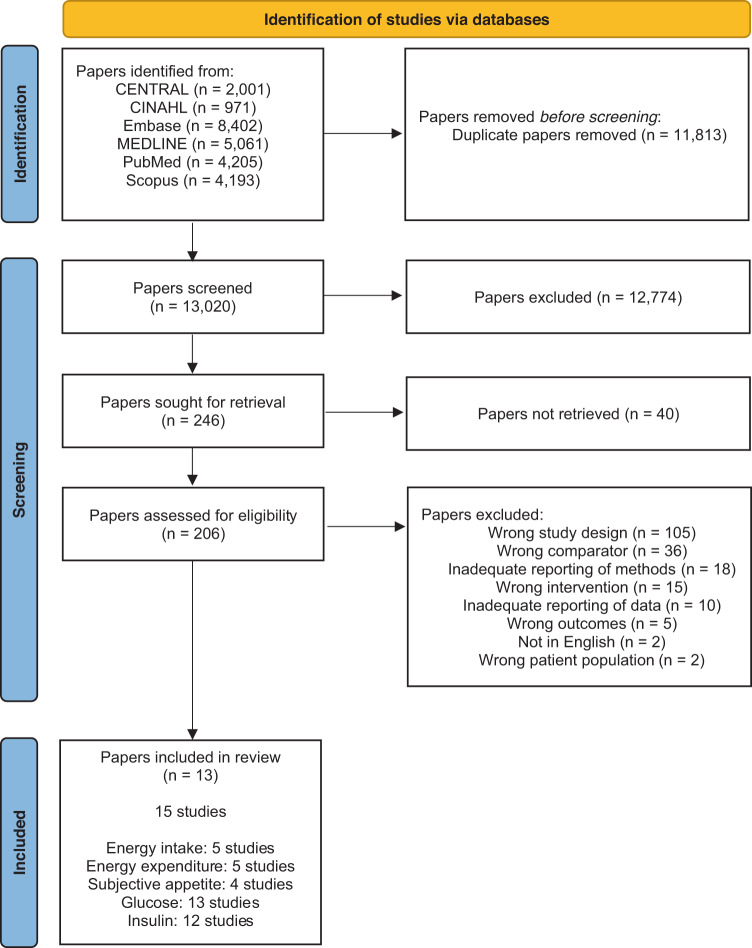
Flow diagram of paper selection. Flow diagram showing the flow of information throught the phases of the systematic review.

Database searching found 24,833 potentially eligible papers. Following removal of duplicates, 13,020 papers underwent title and abstract screening, resulting in the removal of 12,744 papers. Consequently, 246 papers underwent full-text screening, yielding 13 eligible papers. Due to several papers containing multiple studies, a total of 15 separate studies were deemed eligible. The following number of studies proved eligible for each outcome: energy intake, 5 studies; energy expenditure, 5 studies; subjective appetite, 4 studies; glucose, 13 studies; insulin, 12 studies. This process is summarised in Fig. 1.

Several studies appeared to meet inclusion criteria but were subsequently excluded due to insufficient information regarding blinding, randomization, and comparator used (Supplementary Appendix B).

### Study characteristics

Study characteristics of included studies are presented in Table 1.

**Table 1:** Population characteristics, intervention characteristics, and outcome measurements for all included studies.

Study ID	Population				Intervention				Outcomes				
Author	Sample size	Male, %	Age, mean (years)	BMI, mean (kg/m^2^)	Route	Dose	Total dose (mg)	Comparator	Energy intake	Energy expenditure	Subjective appetite	Glucose	Insulin
Arafat et al. [19]^a^	13	46	25.1	21.7	Intramuscular	1 mg bolus at start	1	Saline			✓ (7-point rating scale)	✓ (capillary - glucose oxidase method)	✓ (serum - ELISA)
Arafat et al. [19]^b^	11	45	28.4	34.4	Intramuscular	1 mg bolus at start	1	Saline			✓ (7-point rating scale)	✓ (capillary - glucose oxidase method)	✓ (serum - ELISA)
Bagger et al. [20]	15	100	22.0	23.0	Intravenous	3 ng/kg/min over 270 mins	0.06	Saline	✓ (*ad libitum* meal)	M-IR (indirect calorimetry - face mask)	✓ (VAS)	✓ (plasma - glucose oxidase method)	✓ (serum - ECLIA)
Cegla et al. [45]	13	69	31.6	27.0	Intravenous	9.8 ng/kg/min over 120 mins	0.09	Gelofusine	✓ (*ad libitum* meal)	✓ (indirect calorimetry - canopy)	M-IR (VAS)	✓ (plasma - PL)	✓ (serum - PL)
Chakravarthy et al. [22]	6	100	33.0	23.5	Intravenous	6 ng/kg/min over 600 mins	0.27	Saline		✓ (indirect calorimetry - respiratory chamber)		M-NR (plasma - glucose oxidase method)	M-NR (plasma - ECLIA)
Chernish et al. [49]^a^	12	100	21.0–34.0*	–	Intravenous	2 mg**	2.00	Diluting solution				✓ (plasma - NR)	✓ (plasma - RIA)
Chernish et al. [49]^b^	10	100	22.0–32.0*	–	Intramuscular	2 mg**	2.00	Diluting solution				✓ (plasma - NR)	✓ (plasma - RIA)
Geary et al. [46]	12	100	24.3	21.8	Intravenous	3 ng/kg/min over 10 mins	0.002	Saline	✓ (*ad libitum* meal)				
Izzi-Engbeaya et al. [23]	18	100	25.1	22.5	Intravenous	7 ng/kg/min over 480 mins	0.25	Gelofusine	✓ (*ad libitum* meal)		✓ (VAS)	✓ (capillary - glucose dehydrogenase method)	✓ (serum - PL)
Lockton & Poucher [50]	12	100	42.5	26.0	Intravenous	0.5 mg bolus at start	0.50	Saline				✓ (plasma - NR)	
Ranganath et al. [51]	6	100	23.6	24.0	Intravenous	1.5 mg over 360 mins	1.50	Saline				✓ (plasma - enzymatic method)	✓ (plasma - MEIA)
Salem et al. [21]	11	100	26.1	22.5	Intravenous	50 ng/kg/min over 55 min	0.21	Saline		✓ (indirect calorimetry - canopy)		✓ (plasma - PL)	✓ (plasma - PL)
Schjoldager et al. [52]	9	33	42.0	–	Intravenous	4.9 ng/kg/min over 100 mins	0.04	Saline				✓ (plasma - hexokinase method)	✓ (plasma - RIA)
Stahel et al. [47]	19	79	48.5	29.5	Intranasal	0.7 mg bolus at start	0.70	Sterile dilutant	✓ (*ad libitum* meal)	✓ (indirect calorimetry - face mask)		✓ (plasma - glucose oxidase method)	✓ (plasma - RIA)
Tan et al. [48]	10	70	25.8	29.3	Intravenous	50 ng/kg/min over 45 mins	0.21	Gelofusine		✓ (indirect calorimetry - canopy)		✓ (plasma - PL)	✓ (serum - PL)

^a,b^After author names denotes sub-studies. *only age range provided. **duration of administration not reported. Measurement tools for each outcome are provided in brackets. Note: some doses have been converted to ng from pmol to enable comparisons (ng = pmol ÷ 0.2871).

*ECLIA* electrochemiluminescence immunoassay, *ELISA* enzyme-linked immunosorbent assay, *MEIA* micro particle enzyme immunoassay, *M-IR* measured but inadequately reported, *M-NR* measured but not reported, *NR* not reported, *PL* pathology laboratory, *RIA* radioimmunoassay, *VAS* visual analogue scale.

### Risk of bias

The results of the risk of bias assessment for each outcome are presented in Supplementary Appendix C. With regards to overall risk of bias, there were some concerns for all studies included in the review, irrespective of the outcome measured. This was primarily due to inadequate reporting of the randomization and sequence allocation process, or inadequate reporting of the analysis plan.

### Meta-analysis

Data used for meta-analysis is presented in Supplementary Appendix D. This also includes details of data source.

#### Energy intake

Five studies comprising 77 participants (90% males) measured *ad libitum* energy intake following comparator and glucagon administration [20, 21, 45–47]. Of these five studies, four used intravenous administration [20, 23, 45, 46] and one used intranasal administration [47]. Average age of participants ranged from 22.0 to 48.5 years, with three studies being conducted in healthy-weight participants (18.5 ≥ BMI < 25.0) [20, 23, 46] and two studies being conducted in overweight participants (25.0 ≥ BMI < 30.0) [45, 47].

**Fig. 2:**
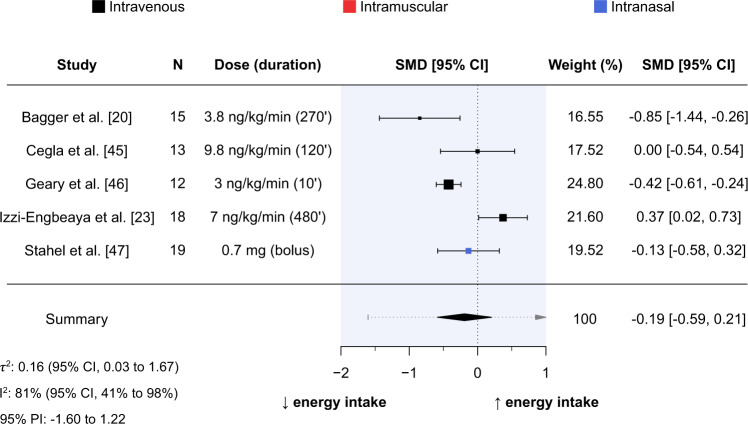
Forest plot of standardised mean differences between glucagon administration and comparator for *ad libitum* energy intake. Results produced from a random-effects meta-analysis using the Hartung-Knapp-Sidik-Jonkman method to estimate between-study variance. Data are presented as mean with 95% confidence intervals. Mean-intervention effect (summary) is also presented alongside a 95% prediction interval (grey horizontal dotted line). Sample size (N), dose (duration of administration in minutes), and route of administration (colour of point estimates) for each study are provided. CI confidence interval, PI prediction interval, SMD standardised mean difference.

Mean intervention effect of glucagon administration relative to comparator on *ad libitum* meal energy intake was SMD = –0.19 (95% CI, –0.59 to 0.21; *P* = 0.345; Fig. 2). Measures of statistical heterogeneity were τ^2^ = 0.16 (95% CI, 0.03–1.67) and I^2^ = 81% (95% CI, 41–98%). According to the 95% PI, the effect size for acute glucagon administration relative to comparator on ad libitum meal energy intake is expected to range from –1.60 to 1.22 in future similar studies. Meta-regression and assessments of publication bias were not performed due to an insufficient number of studies.

#### Energy expenditure

Five studies containing 59 participants (84% males) measured energy expenditure following comparator and glucagon administration [21, 22, 45, 47, 48]. Of these five studies, four used intravenous administration [21, 22, 45, 48] and one used intranasal administration [47]. Average age of participants ranged from 25.8 to 26.1 years, with two studies being conducted in healthy-weight participants [21, 22] and three studies being conducted in overweight participants [45, 47, 48].

**Fig. 3:**
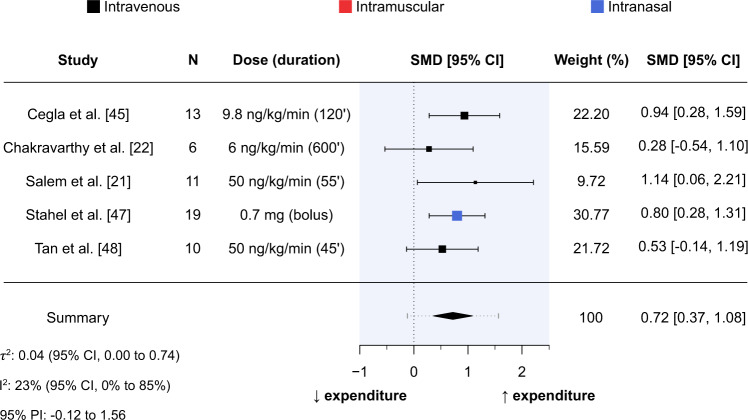
Forest plot of standardised mean differences between glucagon administration and comparator for energy expenditure. Results produced from a random-effects meta-analysis using the Hartung-Knapp-Sidik-Jonkman method to estimate between-study variance. Data are presented as mean with 95% confidence intervals. Mean-intervention effect (summary) is also presented alongside a 95% prediction interval (grey horizontal dotted line). Sample size (N), dose (duration of administration in minutes), and route of administration (colour of point estimates) for each study are provided. CI confidence interval, PI prediction interval, SMD standardised mean difference.

Mean intervention effect of glucagon administration relative to comparator on energy expenditure was SMD = 0.72 (95% CI, 0.37–1.08; *P* < 0.001; Fig. 3). Measures of statistical heterogeneity were τ^2^ = 0.04 (95% CI, 0.00–0.74) and I^2^ = 23% (95% CI, 0–85%). According to the 95% PI, the effect size for acute glucagon administration relative to comparator on energy expenditure is expected to range from –0.12 to 1.56 in future similar studies. Meta-regression and assessments of publication bias were not performed due to an insufficient number of studies.

#### Subjective appetite

Four studies comprising 57 participants (73% males) measured subjective appetite following comparator and glucagon administration [19, 20]. Of these four studies, two used intramuscular [19] administration and two used intravenous administration [20, 23]. Average age of participants ranged from 22.0 to 28.4 years, with three studies being conducted in healthy-weight participants [19, 20, 23] and one study being conducted in obese participants (BMI ≥ 30.0) [19]. Two studies reported subjective satiety [19], one study reported a composite appetite score [20], and one study reported subjective hunger [23].

Due to the limited number of studies, a meta-analysis was not performed. However, two studies reported an increase in subjective appetite following glucagon administration relative to comparator [19, 20] and two studies reported a decrease in subjective appetite [19, 23] (Supplementary Appendix D).

#### Glucose

Thirteen studies comprising 159 participants (80% males) measured glucose following comparator and glucagon administration [19–21, 23, 45, 47–52]. Of these 13 studies, nine used intravenous administration [20, 21, 23, 45, 48–52], 3 used intramuscular administration [19, 49], and one used intranasal administration [47]. Average age of participants ranged from 21.0 to 48.5 years, with five studies being conducted in healthy-weight participants [19–21, 23, 51], four studies being conducted in overweight participants [45, 47, 48, 50], and one study being conducted in obese participants [19]. Three studies did not report participant body weight characteristics [49, 52].

**Fig. 4:**
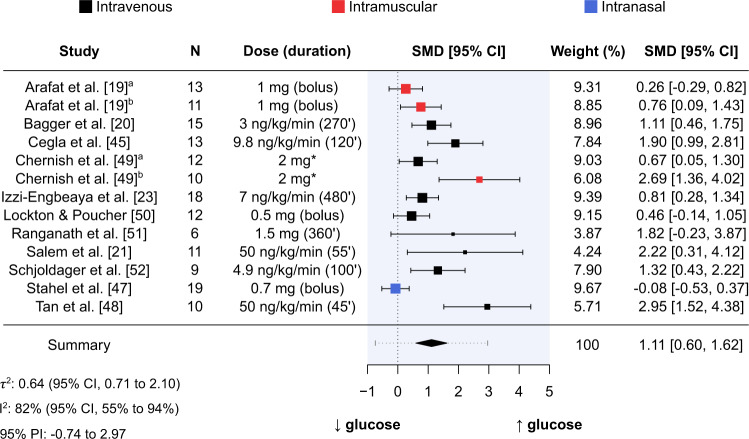
Forest plot of standardised mean differences between glucagon administration and comparator for glucose. Results produced from a random-effects meta-analysis using the Hartung-Knapp-Sidik-Jonkman method to estimate between-study variance. Data are presented as mean with 95% confidence intervals. Mean-intervention effect (summary) is also presented alongside a 95% prediction interval (grey horizontal dotted line). Sample size (N), dose (duration of administration in minutes), and route of administration (colour of point estimates) for each study are provided. *duration of administration not reported. CI confidence interval, PI prediction interval, SMD standardised mean difference.

Mean intervention effect of glucagon administration relative to comparator on glucose was SMD = 1.11 (95% CI, 0.60 to 1.62; *P* < 0.001; Fig. 4). Measures of statistical heterogeneity were τ^2^ = 0.64 (95% CI, 0.71–2.10) and I^2^ = 82% (95% CI, 55–94%). According to the 95% PI, the effect size for acute glucagon administration relative to comparator on glucose is expected to range from –0.74 to 2.97 in future similar studies.

Mixed-effects meta-regression analyses with route of administration or total glucagon dose included as a moderator did not indicate that glucose response differed between administration routes or across total glucagon dose (Supplementary Appendix E).

Visual inspection of the contour-enhanced funnel plot showed asymmetry that indicated potential publication bias (Supplementary Appendix F). This is supported by the result of Egger’s regression test (*P* < 0.001). Trim and fill analysis estimated five missing studies on the left side of the funnel plot (Supplementary Appendix F), resulting in adjusted SMD of 0.63 (95% CI, 0.01, 1.25; *P* = 0.046).

#### Insulin

Twelve studies comprising 147 participants (79% males) measured insulin following comparator and glucagon administration [19–21, 23, 45, 47–49, 51, 52]. Of these 12 studies, eight used intravenous administration [20, 21, 23, 45, 48, 49, 51, 52], three used intramuscular administration [19, 49], and one used intranasal administration [47]. Average age of participants ranged from 21.0 to 48.5, with five studies being conducted in healthy-weight participants [19–21, 23, 51], three studies being conducted in overweight participants [45, 47, 48], and one study being conducted in obese participants [19]. Three studies did not report participant body weight characteristics [49, 52].

**Fig. 5:**
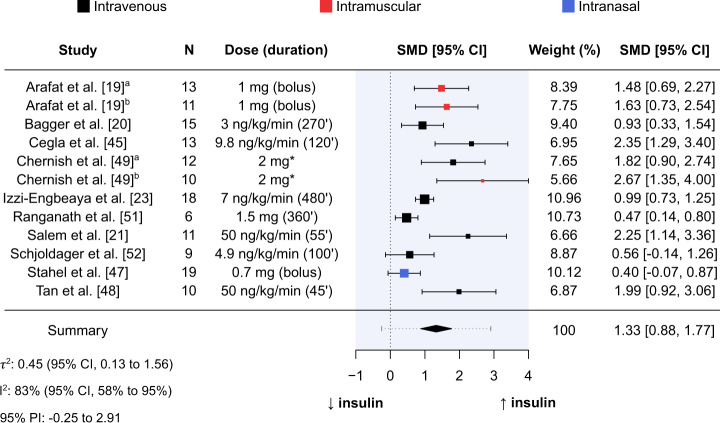
Forest plot of standardised mean differences between glucagon administration and comparator for insulin. Results produced from a random-effects meta-analysis using the Hartung-Knapp-Sidik-Jonkman method to estimate between-study variance. Data are presented as mean with 95% confidence intervals. Mean-intervention effect (summary) is also presented alongside a 95% prediction interval (grey horizontal dotted line). Sample size (N), dose (duration of administration in minutes), and route of administration (colour of point estimates) for each study are provided. *duration of administration not reported. CI confidence interval, PI prediction interval, SMD standardised mean difference.

Mean intervention effect of glucagon administration relative to comparator on insulin was SMD = 1.33 (95% CI, 0.88–1.77; *P* < 0.001; Fig. 5). Measures of statistical heterogeneity were τ^2^ = 0.45 (95% CI, 0.13–1.56) and I^2^ = 83% (95% CI, 58–95%). According to the 95% PI, the effect size for acute glucagon administration relative to comparator on insulin is expected to range from –0.25 to 2.91 in future similar studies.

Mixed-effects meta-regression analyses with route of administration or total glucagon dose included as a moderator did not indicate that insulin response differed between administration routes or across total glucagon dose (Supplementary Appendix E).

Visual inspection of the contour-enhanced funnel plot showed asymmetry that indicated potential publication bias (Supplementary Appendix F). This is supported by the result of Egger’s regression test (*P* < 0.001). Trim and fill analysis estimated three missing studies on the left side of the funnel plot (Supplementary Appendix F), resulting in adjusted SMD of 1.07 (95% CI, 0.57, 1.58; *P* < 0.001).

#### Sensitivity analyses

Sensitivity analyses employing correlation coefficients of 0.3, 0.7 and 0.9 did not meaningfully alter the mean intervention effect and overall interpretation of glucagon administration on energy intake, energy expenditure, glucose, or insulin (Supplementary Appendix G).

Two studies were identified during the full-text screening phase that selected glucagon doses that were defined as sub-anorectic [45] or prevented hyperglycaemia [47]. Consequently, additional sensitivity analyses were performed for energy intake and glucose outcomes excluding these studies from meta-analytical procedures. However, exclusion of these studies did not meaningfully change the mean intervention effect and overall interpretation of results for either outcome (Supplementary Appendix H).

Leave-one-out meta-analyses for energy expenditure, glucose, and insulin did not identify any study that possessed sufficient influence, that when excluded, resulted in a different interpretation of the summary effect estimate (Supplementary Appendix I). The leave-one-out meta-analysis performed for energy intake did identify one study [23] that did exert a noticeable influence, in which its exclusion resulted in a confidence interval (and corresponding P-value) indicative of an anorectic effect (Supplementary Appendix I). However, due to the limited number of studies eligible for this meta-analysis, the importance of this finding is difficult to interpret.

### Certainty of evidence

Certainty of evidence for energy intake, glucose and insulin was rated as low, whilst energy expenditure was rated as high. Explanation of judgements alongside certainty of evidence assessments are presented in the summary of findings table (Table 2).

**Table 2:** Summary of findings.

Outcomes	Relative effect (95% CI)	Number of participants(studies)	Quality of the evidence(GRADE)	Comments
Energy intake	SMD –0.19 decrease with glucagon (–0.59 decrease to 0.21 increase)	77 participants (5 studies)	⊕⊕⊝⊝ Low^a^	
Energy expenditure	SMD 0.72 increase with glucagon (0.37 increase to 1.08 increase)	59 participants (5 studies)	⊕⊕⊕⊕ High	
Glucose	SMD 1.11 increase with glucagon (0.60 increase to 1.62 increase)	159 participants (13 studies)	⊕⊕⊝⊝ Low^b^	Glucose response was not moderated by route of administration or total glucagon dose
Insulin	SMD 1.33 increase with glucagon (0.88 increase to 1.77 increase)	147 participants (12 studies)	⊕⊕⊝⊝ Low^c^	Insulin response was not moderated by route of administration or total glucagon dose

Acute glucagon administration compared with an energy-free control agent in adults without diabetes.

Patient or population: adults without diabetes.

Setting: laboratory environment.

Intervention: acute glucagon administration.

Comparison: energy-free control agent.

GRADE Working Group grades of evidence.

High certainty: we are very confident that the true effect lies close to that of the estimate of the effect.

Moderate certainty: we are moderately confident in the effect estimate: the true effect is likely to be close to the estimate of the effect, but there is a possibility that it is substantially different.

Low certainty: our confidence in the effect estimate is limited: the true effect may be substantially different from the estimate of the effect.

Very low certainty: we have very little confidence in the effect estimate: the true effect is likely to be substantially different from the estimate of effect.

*CI* confidence interval, *SMD* standardised mean difference.

^a^There was considerable heterogeneity (I^2^ = 81.11%; 95% CI, 41.18–97.87%) and 95% confidence interval contained zero. We therefore downgraded by two levels for inconsistency and imprecision.

^b^There was considerable heterogeneity (I^2^ = 82.41%; 95% CI, 55.39–93.88%) that could not be explained by meta-regression. Additionally, asymmetry in the funnel plot and the result of Egger’s regression test suggested possible publication bias. We therefore downgraded by two levels for inconsistency and publication bias.

^c^There was considerable heterogeneity (I^2^ = 83.43%, 95% CI, 58.43–94.55%) that could not be explained by meta-regression. Additionally, asymmetry in the funnel plot and the result of Egger’s regression test suggested possible publication bias. We therefore downgraded by two levels for inconsistency and publication bias.

## Discussion

This review analysed the evidence on the effect of acute glucagon administration on energy intake, energy expenditure, subjective appetite, glucose, and insulin responses in humans. Meta-analyses revealed that, on average, acute glucagon administration increases energy expenditure, as well as glucose and insulin concentrations. However, the effect of acute glucagon administration on energy intake is unclear, whilst too few studies exist to permit a meta-analysis on subjective appetite.

### The effect of acute glucagon administration on components of energy balance

The results from our analysis highlight that the effect of acute glucagon administration on energy intake in humans is inconclusive. Despite the point estimate for the mean intervention effect suggesting a small anorectic effect, the confidence interval for this effect was large and included both an increase and decrease in energy intake following acute glucagon administration. This uncertainty is also reflected in the effect sizes of the individual studies, the prediction interval for energy intake, and the reported effects of acute glucagon administration on subjective appetite. Indeed, no study performed a power calculation based on differences in energy intake between groups (with only two studies stating energy intake as a pre-registered primary outcome [20, 23]), likely contributing to the observed imprecision of the mean intervention effect. The small number of eligible studies also precluded mixed-effects meta-regression analysis from being performed, making it difficult to identify possible moderators responsible for the heterogeneity in responses.

It has been widely reported that glucagon administration can increase feelings of nausea [50, 52–55]. It is therefore possible that any observed effects in energy intake and appetite are secondary to a change in nausea, rather than a direct influence on any appetite-regulation system per se. Subsequently, any inconsistency in response may be related to the degree of nausea induced. However, all authors who assessed nausea reported that glucagon had no effect on the levels of nausea experienced [20, 23, 45], suggesting that nausea is not a likely explanation for the level of inconsistency observed.

Anorectic effects of *chronic* glucagon administration have, however, been previously reported [55, 56]; though a significant decrease in energy intake was only observed after multiple days of administration [55]. This suggests that either acute glucagon doses (<48 hours of continuous infusion) may not suppress energy intake, or that the suppression of energy intake following acute administration may be too small to be detected using the number of participants commonly recruited by studies investigating these effects (<20 participants; Table 1). Both explanations are nonetheless consistent with the findings of the present meta-analysis.

In contrast to its effect on energy intake, the mean intervention effect of acute glucagon administration on energy expenditure was a moderate-to-large increase, which was consistent across all included studies, and congruent with prior research in rodents [12, 57, 58]. Mechanisms responsible for this increase in energy expenditure may include futile substrate cycling, characterised by a repeated conversion between glucose and glucose-6-phosphate [59], but are unlikely related to changes in brown adipose tissue thermogenesis [21]. The absence of any corresponding increase in energy intake of a similar magnitude therefore suggests that acute glucagon administration would have a favourable effect on energy balance in the short-term.

Challenging the acute data on glucagon administration, recent evidence has suggested that chronic administration (72 hours) of glucagon may not raise energy expenditure [55]. This could explain why the smallest effect in our meta-analysis was observed in the study with the longest infusion duration (10 hours) [22], but also questions the efficacy of chronic glucagon administration and/or GCGR receptor agonism for weight loss via increased energy expenditure. Moreover, most studies (3/5 in this review) measuring energy expenditure following acute glucagon administration report pre-post measurements, preventing any investigation of temporal trends (and thus determining the time point at which energy expenditure is no longer raised). However, it remains to be fully elucidated whether the relative conservation of energy expenditure following weight loss would aid weight loss maintenance with a drug that had a glucagonergic element.

Visual inspection of changes in glucose and insulin over time following acute glucagon administration (Supplementary Appendix J) suggest the effect of glucagon on glucose homoeostasis is transient, with values returning to baseline within 200 min of administration. Importantly, insulin and glucose levels return to baseline despite glucagon levels remaining elevated via infusion, implying that acute supraphysiological doses of glucagon do not result in chronically elevated blood glucose concentrations. If it is assumed that the release of stored liver glycogen followed by endogenous insulin release (futile substrate cycling) is the primary driver of increased energy expenditure following acute glucagon administration, then a glucagonergic agent may not have therapeutic benefit as a long-acting preparation. However, if the energy-expenditure rise outlives the acute glucose raising effects (for example, due to effects on amino acid metabolism), then the absence of chronic hyperglycaemia with glucagon treatment is reassuring. Further research is needed to confirm if the increase in energy expenditure following acute glucagon administration is lost when administered chronically, to establish how energy expenditure changes over time following acute administration, and to firmly establish the mechanism by which glucagon acutely and chronically elevates energy expenditure in humans. Given glucagon’s effect on the catecholaminergic system [60], this research should also investigate possible negative effects of chronic glucagon administration such as changes in heart rate and blood pressure.

### The effect of acute glucagon administration on glucose homoeostasis

The mean intervention effect of acute glucagon administration on both glucose and insulin concentrations was large. The effect on glucose concentrations is not surprising considering the well-established role of glucagon in upregulating hepatic glucose production via glycogenolysis [61]. Similarly, glucagon is known to stimulate insulin release both directly (via activation of beta-cells when glucose concentrations are high) and indirectly (via increased glucose concentrations) [62, 63]. Despite most studies reporting an increase in glucose and insulin following acute glucagon administration, the magnitude of this increase varied considerably between studies, resulting in a high degree of statistical heterogeneity that could not be explained by differences in administration route or glucagon dose in our analyses. It is important to note that the number of studies included in the mixed-effects meta-regression analyses (13 and 12 for glucose and insulin outcomes, respectively) was small. Therefore, when considering the small number of studies and large degree of statistical heterogeneity, a moderating effect of administration route or glucagon dose on glucose and insulin responses cannot be excluded as such analyses are likely inadequately powered to detect small to moderate effects [64].

Nevertheless, the consistent increase observed in both insulin and glucose concentrations across studies suggests that they are unlikely to play any potential role in mediating the effects of glucagon on appetite. It is also important to note that the state of hyperglycaemia induced by acute glucagon administration may increase cardiometabolic disease risk [65, 66]. It would therefore seem prudent that any glucagon-based anti-obesity approach is also capable of reducing glucose levels. This could be achieved by a glucagon-like peptide 1 (GLP-1) co-agonist, or a molecule with both GCGR and GLP-1 receptor activity [48].

### Limitations

The present review is subject to several limitations. Firstly, several of the outcomes (energy intake, energy expenditure, and subjective appetite) were only measured by a small number of studies, reducing the precision of summary effect estimates and preventing the use of meta-regression analysis for exploring causes of heterogeneity (e.g. glucagon dose). Secondly, energy intake, glucose, and insulin outcomes showed considerable heterogeneity in response, which was not explained by administration route or total glucagon dose for glucose and insulin outcomes. Owing to the lack of studies to adequately detect moderator effects, it therefore remains unclear what moderates the response of these outcomes following acute glucagon administration. Thirdly, possible publication bias was detected for both glucose and insulin outcomes, suggesting that non-significant findings may have not been published, and that the effect estimates of the present review may be inflated. However, trim-and-fill analyses indicate that that inclusion of theoretical non-significant findings does not change the overall interpretation of the acute effect of glucagon on these outcomes. The possible presence of publication bias alongside large statistical heterogeneity for both glucose and insulin outcomes resulted in the evidence being graded as low certainty. It should be noted that this certainty relates to the precision of the effect estimate, not the direction, as the data clearly shows that acute glucagon administration increases both glucose and insulin concentrations. Fourthly, the participants of included studies were predominantly young (<35 years old) males, with less than half of eligible studies being conducted in participants with a BMI ≥ 25.0. The findings of the present analysis may therefore not be applicable to all populations, particularly those more likely to be treated with anti-obesity agents. Finally, the present analysis only focuses on acute effects of glucagon administration on energy balance and glucose homoeostasis, and thus any observed effects cannot be extrapolated to chronic administration.

### Summary

Overall, acute administration of glucagon in humans appears to produce a marked rise in energy expenditure, glucose, and insulin. However, statistical heterogeneity and potential publication bias reduce our confidence in the effect size estimates for glucose and insulin responses. The effect of acute glucagon administration on energy intake and subjective appetite also remains unclear. Future work should look to clarify the effect of acute glucagon administration on energy intake and appetite, investigate any potential differences between acute and chronic administration, and if needed, develop protocols that can sustain acute beneficial effects such as increased expenditure over longer time periods.

## Supplementary information

PRISMA Checklist

Supplementary Appendix

## Data Availability

Data and R scripts used for analysis are available from the Open Science Framework at: https://osf.io/57xt9/ (10.17605/OSF.IO/57XT9).
