# Supplementary material for: The acute effect of glucagon on components of energy balance and glucose homoeostasis in adults without diabetes: a systematic review and meta-analysis
Source: Int J Obes (Lond). 2022 Sep 19;46(11):1948–59. doi: 10.1038/s41366-022-01223-y (PMC9584822; doi:10.1038/s41366-022-01223-y)
Supplement: Supplementary file 2 — Supplementary Appendix [file 41366_2022_1223_MOESM2_ESM.docx]

**Supplementary Appendix A:** Search strategy

**CENTRAL**

#1 (glucagon):ti,ab,kw AND (infus* OR admin* OR intravenous OR dos* OR saline OR subcutaneous OR intramuscular OR nasal):ti,ab,kw AND (energy OR intake OR food OR meal OR appetite OR eat* OR hunger OR hungry OR expenditure OR calorie* OR kcal OR joule* OR kJ OR insulin OR glucose OR glyc*):ti,ab,kw AND (human* OR participant* OR subject* OR patient* OR volunteer* OR men OR women OR man OR woman OR male* OR female* OR individual* OR recruit* OR trial OR crossover OR cross over OR cross-over OR consent OR assign* OR allocate* OR placebo):ti,ab,kw

#2 (review OR meta analysis OR meta-analysis OR case study OR case studies):ti OR (databases adj4 searched):ab OR (rat OR rats or mouse OR mice OR swine OR porcine OR murine OR sheep OR lamb OR pigs OR piglets OR rabbit OR rabbits OR cat OR cats OR dog OR dogs OR cattle OR bovine OR monkey OR monkeys OR trout OR chick OR chicks OR broiler OR broilers OR carp):ti

#3 MeSH descriptor: [Animals] in all MeSH products

#4 MeSH descriptor: [Models, Animal] explode all trees

#5 #1 NOT #2 NOT #3 NOT #4"

**CINAHL**

"AB glucagon

AND AB (infus* OR admin* OR intravenous OR dos* OR saline OR subcutaneous OR intramuscular OR nasal)

AND AB (energy OR intake OR food OR meal OR appetite OR eat* OR hunger OR hungry OR expenditure OR calorie* OR kcal OR joule* OR kJ OR insulin OR glucose OR glyc*)

AND AB (human* OR participant* OR subject* OR patient* OR volunteer* OR men OR women OR man OR woman OR male* OR female* OR individual* OR recruit* OR trial OR crossover OR cross over OR cross-over OR consent OR assign* OR allocate* OR placebo)

NOT TI (review OR meta analysis OR meta-analysis OR case study OR case studies)

NOT AB databases adj4 searched NOT TI (rat OR rats or mouse OR mice OR swine OR porcine OR murine OR sheep OR lamb OR pigs OR piglets OR rabbit OR rabbits OR cat OR cats OR dog OR dogs OR cattle OR bovine OR monkey OR monkeys OR trout OR chick OR chicks OR broiler OR broilers OR carp)

NOT MW (animal* OR animal studies OR animal model*)"

**Embase**

1. glucagon.ab,kw,ti.

2. (infus* or admin* or intravenous or dos* or saline or subcutaneous or intramuscular or nasal).ab,kw,ti.

3. (energy or intake or food or meal or appetite or eat* or hunger or hungry or expenditure or calorie* or kcal or joule* or kJ or insulin or glucose or glyc*).ab,kw,ti.

4. (human* or participant* or subject* or patient* or volunteer* or men or women or man or woman or male* or female* or individual* or recruit* or trial or crossover or cross over or cross-over or consent or assign* or allocate* or placebo).ab,kw,ti.

5. (review or meta analysis or meta-analysis or case study).ti.

6. (databases adj4 searched).ab.

7. (rat or rats or mouse or mice or swine or porcine or murine or sheep or lamb or pigs or piglets or rabbit or rabbits or cat or cats or dog or dogs or cattle or bovine or monkey or monkeys or trout or chick or chicks or broiler or broilers or carp).ti.

8. (animal* or animal studies or animal model*).sh.

9. (1 and 2 and 3 and 4) not 5 not 6 not 7 not 8

**Medline**

1. glucagon.ab,kw,ti.

2. (infus* or admin* or intravenous or dos* or saline or subcutaneous or or intramuscular or nasal).ab,kw,ti.

3. (energy or intake or food or meal or appetite or eat* or hunger or hungry or expenditure or calorie* or kcal or joule* or kJ or insulin or glucose or glyc*).ab,kw,ti.

4. (human* or participant* or subject* or patient* or volunteer* or men or women or man or woman or male* or female* or individual* or recruit* or trial or crossover or cross over or cross-over or consent or assign* or allocate* or placebo).ab,kw,ti.

5. (review or meta analysis or meta-analysis or case study).ti.

6. (databases adj4 searched).ab.

7. (rat or rats or mouse or mice or swine or porcine or murine or sheep or lamb or pigs or piglets or rabbit or rabbits or cat or cats or dog or dogs or cattle or bovine or monkey or monkeys or trout or chick or chicks or broiler or broilers or carp).ti.

8. (animal* or animal studies or animal model*).sh.

9. (1 and 2 and 3 and 4) not 5 not 6 not 7 not 8

**PubMed**

(((((((glucagon[Title/Abstract])

AND (infus*[Title/Abstract] OR admin*[Title/Abstract] OR intravenous[Title/Abstract] OR dos*[Title/Abstract] OR saline[Title/Abstract] OR subcutaneous[Title/Abstract] OR intramuscular[Title/Abstract] OR nasal[Title/Abstract]))

AND (energy[Title/Abstract] OR intake[Title/Abstract] OR food[Title/Abstract] OR meal[Title/Abstract] OR appetite[Title/Abstract] OR eat*[Title/Abstract] OR hunger[Title/Abstract] OR hungry[Title/Abstract] OR expenditure[Title/Abstract] OR calorie*[Title/Abstract] OR kcal[Title/Abstract] OR joule*[Title/Abstract] OR kJ[Title/Abstract] OR insulin[Title/Abstract] OR glucose[Title/Abstract] OR glyc*[Title/Abstract]))

AND (human*[Title/Abstract] OR participant*[Title/Abstract] OR subject*[Title/Abstract] OR patient*[Title/Abstract] OR volunteer*[Title/Abstract] OR men[Title/Abstract] OR women[Title/Abstract] OR man[Title/Abstract] OR woman[Title/Abstract] OR male*[Title/Abstract] OR female*[Title/Abstract] OR individual*[Title/Abstract] OR recruit*[Title/Abstract] OR trial[Title/Abstract] OR crossover[Title/Abstract] OR cross over[Title/Abstract] OR cross-over[Title/Abstract] OR consent[Title/Abstract] OR assign*[Title/Abstract] OR allocate*[Title/Abstract] OR placebo[Title/Abstract]))

NOT (review[Title] OR meta analysis[Title] OR meta-analysis[Title] OR case study[Title] OR case studies[Title]))

NOT (databases adj4 searched[Title/Abstract]))

NOT (rat[Title] OR rats[Title] OR mouse[Title] OR mice[Title] OR swine[Title] OR porcine[Title] OR murine[Title] OR sheep[Title] OR lamb[Title] OR pigs[Title] OR piglets[Title] OR rabbit[Title] OR rabbits[Title] OR cat[Title] OR cats[Title] OR dog[Title] OR dogs[Title] OR cattle[Title] OR bovine[Title] OR monkey[Title] OR monkeys[Title] OR trout[Title] OR chick[Title] OR chicks[Title] OR broiler[Title] OR broilers[Title] OR carp[Title]))

NOT (animal* OR animal studies OR animal model*[MeSH Terms])

**Scopus**

(TITLE-ABS-KEY (glucagon)

AND TITLE-ABS-KEY (infus* OR admin* OR intravenous OR dos* OR saline OR subcutaneous OR intramuscular OR nasal)

AND TITLE-ABS-KEY (energy OR intake OR food OR meal OR appetite OR eat* OR hunger OR hungry OR expenditure OR calorie* OR kcal OR joule* OR kj OR insulin OR glucose OR glyc*)

AND TITLE-ABS-KEY (human* OR participant* OR subject* OR patient* OR volunteer* OR men OR women OR man OR woman OR male* OR female* OR individual* OR recruit* OR trial OR crossover OR cross AND over OR cross-over OR consent OR assign* OR allocate* OR placebo)

AND NOT TITLE (review OR meta AND analysis OR meta-analysis OR case AND study OR case AND studies)

AND NOT ABS (databases AND adj4 AND searched)

AND NOT TITLE (rat OR rats OR mouse OR mice OR swine OR porcine OR murine OR sheep OR lamb OR pigs OR piglets OR rabbit OR rabbits OR cat OR cats OR dog OR dogs OR cattle OR bovine OR monkey OR monkeys OR trout OR chick OR chicks OR broiler OR broilers OR carp))

**Supplementary Appendix B:** Characteristics of excluded studies

| **Study** | **Reason for exclusion** | **Outcomes measured** |
| --- | --- | --- |
| Alford et al. ^1^ | Does not state nature of blinding | Glucose, insulin |
| Arvat et al. ^2^ | Does not state if study is randomized; does not state nature of blinding | Glucose |
| Breckenridge et al. ^3^ | Does not state nature of blinding | Glucose, insulin |
| Broglio et al. ^4^ | Does not state nature of blinding | Glucose, insulin |
| Chang et al. ^5^ | Does not state nature of blinding | Glucose |
| Cremer et al. ^6^ | Does not state if study is randomized; does not state nature of blinding | Glucose, insulin |
| Greco et al. ^7^ | Does not state if study is randomized; does not state nature of blinding | Glucose, insulin |
| Kabadi & Premachandra ^8^ | Does not state nature of blinding | Glucose, insulin |
| Larsen et al. ^9^ | Does not state comparator used | Glucose, insulin |
| Liljenquist & Rabin ^10^ | Does not state if study is randomized; does not state nature of blinding | Glucose, insulin |
| Massara et al. ^11^ | Does not state nature of blinding | Glucose, insulin |
| Meier et al. ^12^ | Does not state nature of blinding | Glucose, insulin |
| Penick et al. ^13^ | Does not state comparator used | Energy intake |
| Pontiroli et al. ^14^ | Does not state nature of blinding | Glucose, insulin |
| Schade & Eaton ^15^ | Does not state nature of blinding | Glucose, insulin |
| Schade & Eaton ^16^ | Does not state nature of blinding | Glucose, insulin |
| Sherwin et al. ^17^ | Does not state nature of blinding | Glucose, insulin |
| Turner et al. ^18^ | Does not state if study is randomized; does not state nature of blinding; does not state comparator used | Glucose, insulin |

Attempts were made to contact study authors to clarify methods used and determine study eligibility. Authors either did not respond or provided sufficient information to include or exclude the study. However, for several studies, contact information could not be obtained ^6,10,11,13^.

**Supplementary Appendix C:** Risk of bias analysis

**Energy intake**

| **Study** | **Bias arising from the randomization process** | **Bias arising from period and carryover effects** | **Bias due to deviations from intended intervention** | **Bias due to missing outcome data** | **Bias in the measurement of the outcome** | **Bias in the selection of the reported result** | **Overall risk of bias** |
| --- | --- | --- | --- | --- | --- | --- | --- |
| Bagger et al. ^19^ | Some concerns | Some concerns | Low risk | Low risk | Low risk | Low risk | Some concerns |
| Cegla et al. ^20^ | Some concerns | Some concerns | Low risk | Low risk | Low risk | Some concerns | Some concerns |
| Geary et al. ^21^ | Some concerns | Low risk | Low risk | Low risk | Low risk | Some concerns | Some concerns |
| Izzi-Engbeaya et al. ^22^ | Some concerns | Some concerns | Low risk | Low risk | Low risk | Low risk | Some concerns |
| Stahel et al. ^23^ | Some concerns | Low risk | Low risk | Low risk | Low risk | Low risk | Some concerns |

**Energy expenditure**

| **Study** | **Bias arising from the randomization process** | **Bias arising from period and carryover effects** | **Bias due to deviations from intended intervention** | **Bias due to missing outcome data** | **Bias in the measurement of the outcome** | **Bias in the selection of the reported result** | **Overall risk of bias** |
| --- | --- | --- | --- | --- | --- | --- | --- |
| Cegla et al. ^20^ | Some concerns | Some concerns | Low risk | Low risk | Low risk | Some concerns | Some concerns |
| Chakravarthy et al. ^24^ | Some concerns | Low risk | Low risk | Low risk | Low risk | Low risk | Some concerns |
| Salem et al. ^25^ | Some concerns | Some concerns | Low risk | Low risk | Low risk | Low risk | Some concerns |
| Stahel et al. ^23^ | Some concerns | Low risk | Low risk | Low risk | Low risk | Low risk | Some concerns |
| Tan et al. ^26^ | Some concerns | Low risk | Low risk | Low risk | Low risk | Some concerns | Some concerns |

**Appetite**

| **Study** | **Bias arising from the randomization process** | **Bias arising from period and carryover effects** | **Bias due to deviations from intended intervention** | **Bias due to missing outcome data** | **Bias in the measurement of the outcome** | **Bias in the selection of the reported result** | **Overall risk of bias** |
| --- | --- | --- | --- | --- | --- | --- | --- |
| Arafat et al. ^27a^ | Some concerns | Some concerns | Low risk | Low risk | Low risk | Some concerns | Some concerns |
| Arafat et al. ^27b^ | Some concerns | Some concerns | Low risk | Low risk | Low risk | Some concerns | Some concerns |
| Bagger et al. ^19^ | Some concerns | Some concerns | Low risk | Low risk | Low risk | Low risk | Some concerns |
| Izzi-Engbeaya et al. ^22^ | Some concerns | Some concerns | Low risk | Low risk | Low risk | Low risk | Some concerns |

**Glucose**

| **Study** | **Bias arising from the randomization process** | **Bias arising from period and carryover effects** | **Bias due to deviations from intended intervention** | **Bias due to missing outcome data** | **Bias in the measurement of the outcome** | **Bias in the selection of the reported result** | **Overall risk of bias** |
| --- | --- | --- | --- | --- | --- | --- | --- |
| Arafat et al. ^27a^ | Some concerns | Some concerns | Low risk | Low risk | Low risk | Some concerns | Some concerns |
| Arafat et al. ^27b^ | Some concerns | Some concerns | Low risk | Low risk | Low risk | Some concerns | Some concerns |
| Bagger et al. ^19^ | Some concerns | Some concerns | Low risk | Low risk | Low risk | Some concerns | Some concerns |
| Cegla et al. ^20^ | Some concerns | Some concerns | Low risk | Low risk | Low risk | Some concerns | Some concerns |
| Chernish et al. ^28a^ | Some concerns | Low risk | Low risk | Low risk | Low risk | Some concerns | Some concerns |
| Chernish et al. ^28b^ | Some concerns | Low risk | Low risk | Low risk | Low risk | Some concerns | Some concerns |
| Izzi-Engbeaya et al. ^22^ | Some concerns | Some concerns | Low risk | Low risk | Low risk | Low risk | Some concerns |
| Lockton & Poucher ^29^ | Some concerns | Low risk | Low risk | Low risk | Low risk | Low risk | Some concerns |
| Ranganath et al. ^30^ | Some concerns | Some concerns | Low risk | Low risk | Low risk | Some concerns | Some concerns |
| Salem et al. ^25^ | Some concerns | Some concerns | Low risk | Low risk | Low risk | Some concerns | Some concerns |
| Schjoldager et al. ^31^ | Some concerns | Some concerns | Low risk | Low risk | Low risk | Some concerns | Some concerns |
| Stahel et al. ^23^ | Some concerns | Low risk | Low risk | Low risk | Low risk | Low risk | Some concerns |
| Tan et al. ^26^ | Some concerns | Low risk | Low risk | Low risk | Low risk | Some concerns | Some concerns |

**Insulin**

| **Study** | **Bias arising from the randomization process** | **Bias arising from period and carryover effects** | **Bias due to deviations from intended intervention** | **Bias due to missing outcome data** | **Bias in the measurement of the outcome** | **Bias in the selection of the reported result** | **Overall risk of bias** |
| --- | --- | --- | --- | --- | --- | --- | --- |
| Arafat et al. ^27^ | Some concerns | Some concerns | Low risk | Low risk | Low risk | Some concerns | Some concerns |
| Arafat et al. ^27^ | Some concerns | Some concerns | Low risk | Low risk | Low risk | Some concerns | Some concerns |
| Bagger et al. ^19^ | Some concerns | Some concerns | Low risk | Low risk | Low risk | Some concerns | Some concerns |
| Cegla et al. ^20^ | Some concerns | Some concerns | Low risk | Low risk | Low risk | Some concerns | Some concerns |
| Chernish et al. ^28a^ | Some concerns | Low risk | Low risk | Low risk | Low risk | Some concerns | Some concerns |
| Chernish et al. ^28b^ | Some concerns | Low risk | Low risk | Low risk | Low risk | Some concerns | Some concerns |
| Izzi-Engbeaya et al. ^22^ | Some concerns | Some concerns | Low risk | Low risk | Low risk | Low risk | Some concerns |
| Ranganath et al. ^30^ | Some concerns | Some concerns | Low risk | Low risk | Low risk | Some concerns | Some concerns |
| Salem et al. ^25^ | Some concerns | Some concerns | Low risk | Low risk | Low risk | Some concerns | Some concerns |
| Schjoldager et al. ^31^ | Some concerns | Some concerns | Low risk | Low risk | Low risk | Some concerns | Some concerns |
| Stahel et al. ^23^ | Some concerns | Low risk | Low risk | Low risk | Low risk | Low risk | Some concerns |
| Tan et al. ^26^ | Some concerns | Low risk | Low risk | Low risk | Low risk | Some concerns | Some concerns |

Note: Answers to signalling questions and explanations of judgements for all outcomes are provided at: <https://osf.io/57xt9/> (DOI: 10.17605/OSF.IO/57XT9)

**Supplementary Appendix D:** Data used for meta-analysis

**Energy intake**

| **Author** | **Sample  size** | **Comparator** | | **Glucagon** | | **Data source** |
| --- | --- | --- | --- | --- | --- | --- |
|  |  | **Mean** | **SD** | **Mean** | **SD** |  |
| Bagger et al. ^19^ | 15 | 811 | 147 | 686 | 148 | Primary reference (Table 2) |
| Cegla et al. ^20^ | 13 | 1086 | 397 | 1086 | 349 | Primary reference (In text) |
| Geary et al. ^21^ | 12 | 803 | 314 | 672 | 302 | Primary reference (Table 4) |
| Izzi-Engbeaya et al. ^22^ | 17 | 1069 | 371 | 1213 | 402 | Correspondence with authors |
| Stahel et al. ^23^ | 19 | 1206 | 309 | 1165 | 309 | Primary reference (In text) |

**Energy expenditure**

| **Author** | **Sample  size** | **Control** | | **Glucagon** | | **Data source** |
| --- | --- | --- | --- | --- | --- | --- |
|  |  | **Mean** | **SD** | **Mean** | **SD** |  |
| Cegla et al. ^20^ | 13 | -9 | 45 | 67 | 106 | Primary reference (Figure 5A) - extracted using WebPlotDigitizer |
| Chakravarthy et al. ^24^ | 6 | 13750 | 1472 | 14153 | 1403 | Primary reference (Figure 5A) - extracted using WebPlotDigitizer and AUC calculated |
| Salem et al. ^25^ | 11 | 64 | 163 | 214 | 89 | Correspondence with authors |
| Stahel et al. ^23^ | 19 | 62 | 10 | 69 | 10 | Primary reference (Figure 3A) - extracted using WebPlotDigitizer |
| Tan et al. ^26^ | 10 | Only mean difference provided | | Only mean difference provided | | Primary reference (Table 2) |

AUC, area under the curve.

**Subjective appetite**

| **Author** | **Sample  size** | **Control** | | **Glucagon** | | **Data source** |
| --- | --- | --- | --- | --- | --- | --- |
|  |  | **Mean** | **SD** | **Mean** | **SD** |  |
| Arafat et al. ^27a^ | 13 | 189* | 62 | 284* | 50 | Primary reference (Figure 1D) - extracted using WebPlotDigitizer |
| Arafat et al. ^27b^ | 11 | 274* | 125 | 212* | 33 | Primary reference (Figure 1D) - extracted using WebPlotDigitizer |
| Bagger et al. ^19^ | 15 | 46 | 33 | 57 | 33 | Primary reference (Table 2) |
| Izzi-Engbeaya et al. [22] | 16 | 3110 | 795 | 2865 | 719 | Correspondence with authors |

*satiety score measured (inverse of appetite)

**Glucose**

| **Author** | **Sample  size** | **Control** | | **Glucagon** | | **Data source** |
| --- | --- | --- | --- | --- | --- | --- |
|  |  | **Mean** | **SD** | **Mean** | **SD** |  |
| Arafat et al. ^27a^ | 13 | 23175 | 1335 | 24077 | 4645 | Primary reference (Figure 4A) - extracted using WebPlotDigitizer and AUC calculated |
| Arafat et al. ^27b^ | 11 | 23550 | 3041 | 26315 | 4159 | Primary reference (Figure 4C) - extracted using WebPlotDigitizer and AUC calculated |
| Bagger et al. ^19^ | 15 | 1234 | 66 | 1307 | 66 | Primary reference (Table 1) |
| Cegla et al. ^20^ | 13 | 425 | 45 | 533 | 67 | Primary reference (Figure 2C) - extracted using WebPlotDigitizer |
| Chernish et al.^28a^ | 12 | 215 | 17 | 23 | 33 | Primary reference (Table 1) |
| Chernish et al.^28b^ | 10 | 213 | 10 | 284 | 36 | Primary reference (Table 3) |
| Izzi-Engbeaya et al. ^22^ | 18 | 2450 | 276 | 2687 | 308 | Primary reference (Figure 1C) - extracted using WebPlotDigitizer |
| Lockton & Poucher ^29^ | 12 | 923 | 49 | 951 | 71 | Primary reference (Figure 1) - extracted using WebPlotDigitizer and AUC calculated |
| Ranganath et al. ^30^ | 6 | 2294 | 112 | 2675 | 273 | Correspondence with authors |
| Salem et al. ^25^ | 11 | 574 | 46 | 713 | 76 | Correspondence with authors |
| Schjoldager et al. ^31^ | 9 | 429 | 62 | 536 | 97 | Primary reference (Figure 3) - extracted using WebPlotDigitizer and AUC calculated |
| Stahel et al. ^23^ | 19 | 90 | 9 | 90 | 10 | Primary reference (Figure 2C) - extracted using WebPlotDigitizer |
| Tan et al. ^26^ | 10 | 381 | 25 | 513 | 58 | Primary reference (Figure 2B) - extracted using WebPlotDigitizer |

AUC, area under the curve.

**Insulin**

| **Author** | **Sample  size** | **Control** | | **Glucagon** | | **Data source** |
| --- | --- | --- | --- | --- | --- | --- |
|  |  | **Mean** | **SD** | **Mean** | **SD** |  |
| Arafat et al. ^27a^ | 13 | 20898 | 9346 | 43426 | 19357 | Primary reference (Figure 4B) - extracted using WebPlotDigitizer and AUC calculated |
| Arafat et al. ^27b^ | 11 | 21276 | 11008 | 52645 | 24826 | Primary reference (Figure 4D) - extracted using WebPlotDigitizer and AUC calculated |
| Bagger et al. ^19^ | 15 | 35 | 9 | 43 | 8 | Primary reference (Table 1) |
| Cegla et al. ^20^ | 13 | 587 | 372 | 2225 | 914 | Primary reference (Figure 2D) - extracted using WebPlotDigitizer |
| Chernish et al. ^28a^ | 12 | 38 | 9 | 89 | 38 | Primary reference (Table 1) |
| Chernish et al.^28b^ | 10 | 35 | 11 | 152 | 61 | Primary reference (Table 3) |
| Izzi-Engbeaya et al. ^22^ | 18 | 2527 | 1361 | 4147 | 1866 | Correspondence with authors |
| Ranganath et al. ^30^ | 6 | 36010 | 40071 | 59984 | 59810 | Correspondence with authors |
| Salem et al. ^25^ | 11 | 547 | 442 | 2616 | 1223 | Primary reference (Figure S2C) - extracted using WebPlotDigitizer and AUC calculated |
| Schjoldager et al. ^31^ | 9 | 1247 | 1147 | 1931 | 1285 | Primary reference (Figure 3) - extracted using WebPlotDigitizer and AUC calculated |
| Stahel et al. ^23^ | 19 | 71 | 59 | 98 | 77 | Primary reference (Figure 2B) - extracted using WebPlotDigitizer |
| Tan et al. ^26^ | 10 | 513 | 325 | 2921 | 1682 | Primary reference (Figure 2D) - extracted using WebPlotDigitizer |

AUC, area under the curve.

NOTE: Sample sizes reported in Supplementary Appendix 4 may differ from those reported in corresponding manuscripts. This is because individual participant data was obtained for these studies, but this data contained missing values for some participants.

**Supplementary Appendix E:** Mixed-effects meta-regression model results

**Route of administration**

Administration routes were coded as follows: intravenous (0); intramuscular (1), intranasal (2). Therefore, coefficients for intramuscular and intranasal routes are relative to the intercept (intravenous).

**Glucose**

Omnibus test of moderator coefficients: P = 0.292

I^2^ (original model): 82% (95% CI, 55% to 94%)

I^2^ (route of administration included as a moderator): 79% (95% CI, 39% to 93%).

|  | **Estimate** | **Standard error** | **t-value** | **P-value** | **95% Confidence interval** |
| --- | --- | --- | --- | --- | --- |
| Intercept (intravenous) | 1.30 | 0.30 | 4.385 | 0.001 | 0.64, 1.96 |
| Intramuscular | -0.26 | 0.57 | -0.445 | 0.666 | -1.53, 1.02 |
| Intranasal | -1.38 | 0.83 | -1.666 | 0.127 | -3.22, 0.47 |

**Insulin**

Omnibus test of moderator coefficients: P = 0.237

I^2^ (original model): 83% (95% CI, 58% to 95%)

I^2^ (route of administration included as a moderator): 81% (95% CI, 44% to 94%)

|  | **Estimate** | **Standard error** | **t-value** | **P-value** | **95% Confidence interval** |
| --- | --- | --- | --- | --- | --- |
| Intercept (intravenous) | 1.29 | 0.25 | 5.149 | <0.001 | 0.72, 1.86 |
| Intramuscular | 0.55 | 0.51 | 1.074 | 0.311 | -0.61, 1.71 |
| Intranasal | -0.89 | 0.69 | -1.283 | 0.232 | -2.45, 0.68 |

**Dose**

Total glucagon doses for each study were calculated and converted to a common unit of measurement (ng per kg). For studies that did not provide the glucagon dose relative to body mass, and also did not provide mean participant body mass, a body mass of 75 kg was assumed in order to convert dosage to ng per kg.

**Glucose**

I^2^ (original model): 82% (95% CI, 55% to 94%)

I^2^ (total glucagon dose included as a moderator): 83% (95% CI, 58% to 95%)

|  | **Estimate** | **Standard error** | **t-value** | **P-value** | **95% Confidence interval** |
| --- | --- | --- | --- | --- | --- |
| Intercept | 1.18 | 0.38 | 3.101 | 0.010 | 0.34, 2.02 |
| Total glucagon dose | -0.00 | 0.00 | -0.244 | 0.827 | -0.00, 0.00 |


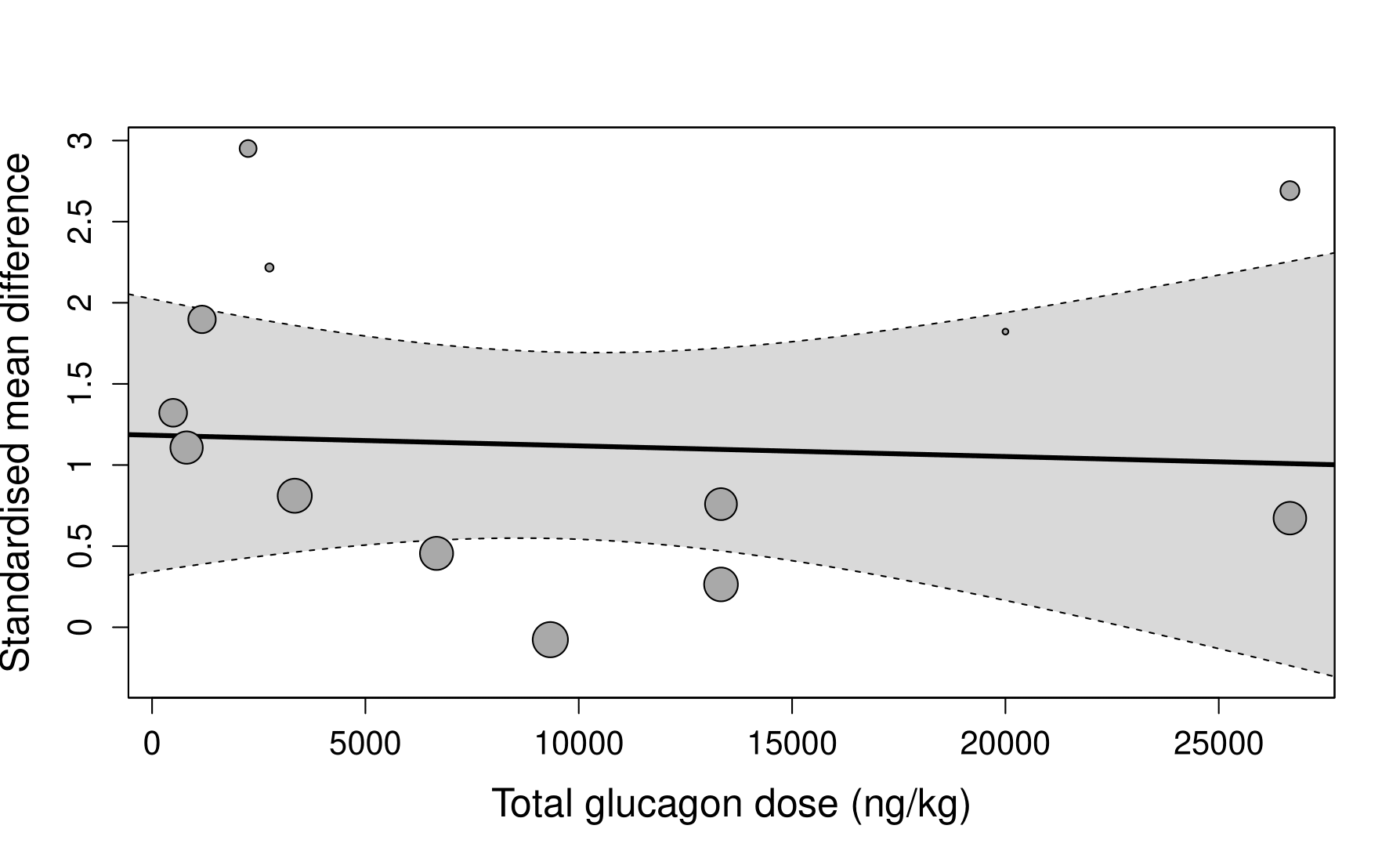


**Figure S1:** Bubble plot of standardised mean difference for glucose against total glucagon dose. Circles represent individual studies, with the size of circle proportional to the weight assigned in the analysis. Dark line represents predicted standardised mean difference as a function of total glucagon dose, with corresponding 95% confidence interval bounds (shaded grey area).

**Insulin**

I^2^ (original model): 83% (95% CI, 58% to 95%)

I^2^ (total glucagon dose included as a moderator): 82% (95% CI, 58% to 94%)

|  | **Estimate** | **Standard error** | **t-value** | **P-value** | **95% Confidence interval** |
| --- | --- | --- | --- | --- | --- |
| Intercept | 1.22 | 0.33 | 3.66 | 0.004 | 0.48, 1.96 |
| Total glucagon dose | 0.00 | 0.00 | 0.47 | 0.646 | -0.00, 0.00 |


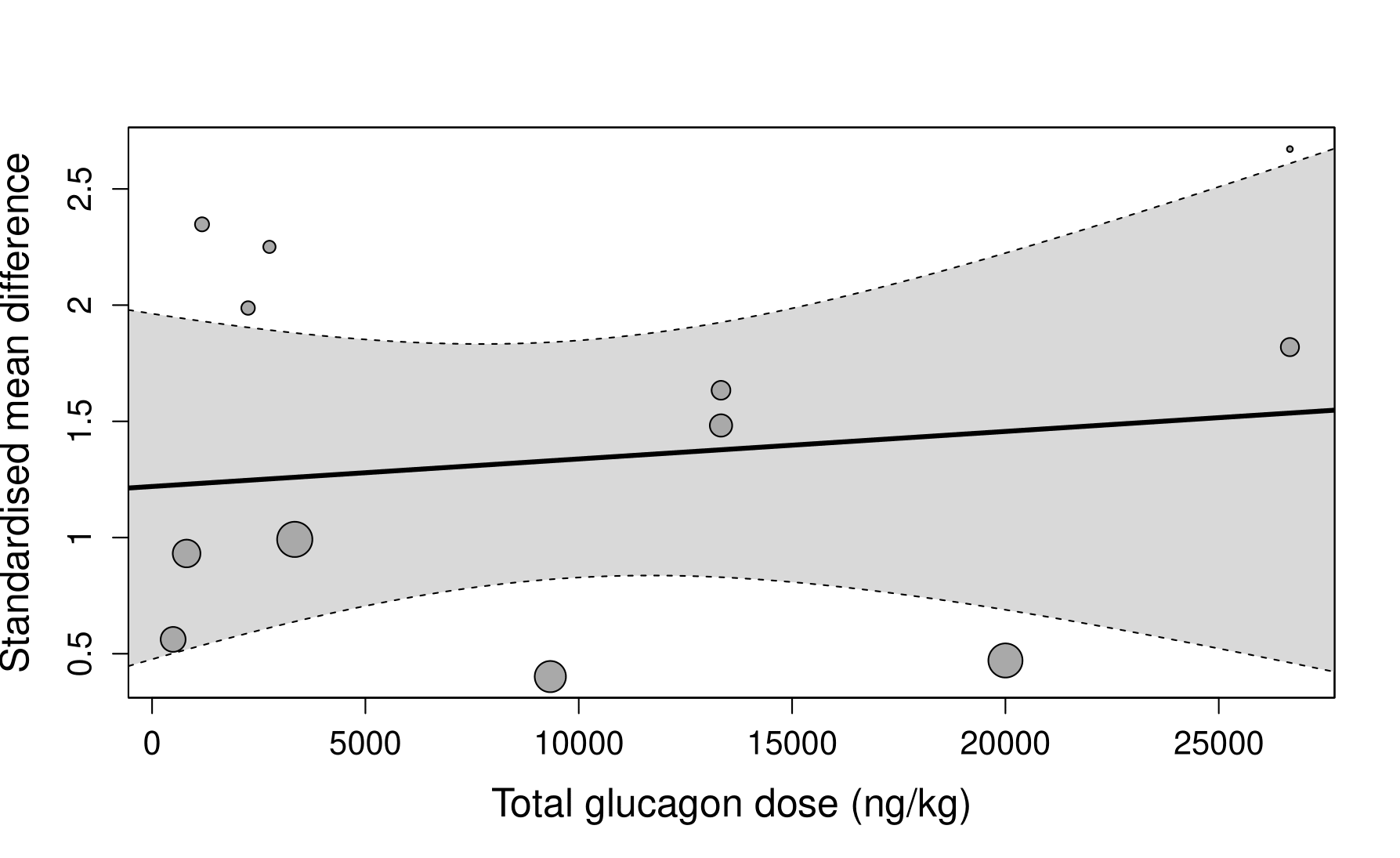


**Figure S2:** Bubble plot of standardised mean difference for insulin against total glucagon dose. Circles represent individual studies, with the size of circle proportional to the weight assigned in the analysis. Dark line represents predicted standardised mean difference as a function of total glucagon dose, with corresponding 95% confidence interval bounds (shaded grey area).

**Supplementary Appendix F:** Contour-enhanced funnel plots

**Glucose**


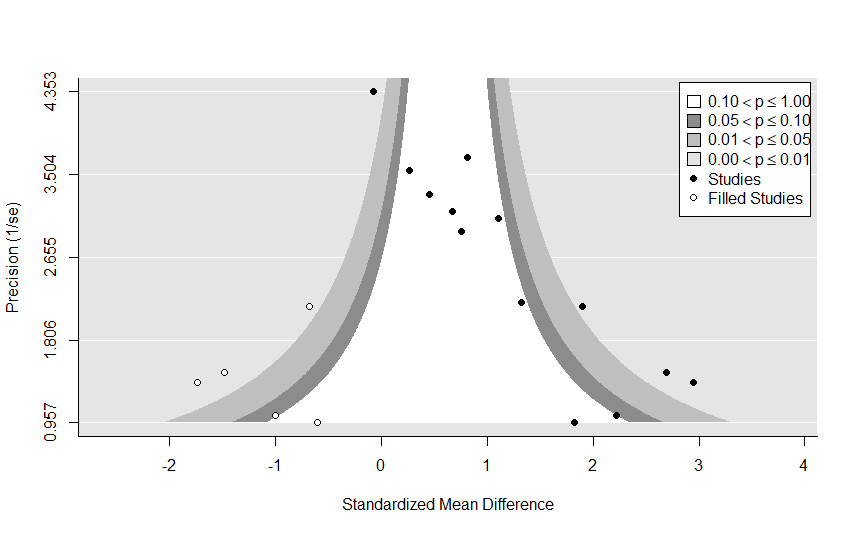

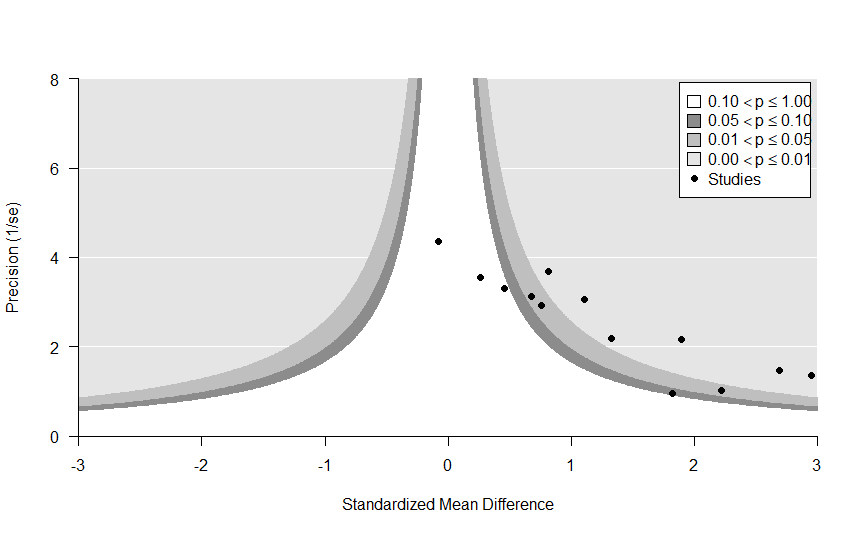


**Figure S4:** Contour-enhanced funnel plot for studies measuring glucose following trim and fill analysis.

**Figure S3:** Contour-enhanced funnel plot for studies measuring glucose.

| **Model** | **Mean intervention effect** | **95% confidence interval** | **95% prediction interval** | **P-value** | **Tau-squared [95% CI]** | **I-squared [95% CI]** |
| --- | --- | --- | --- | --- | --- | --- |
| Original | 1.11 | 0.60, 1.62 | -0.74, 2.97 | <0.001 | 0.64 [0.71, 2.10] | 82 [55, 94] |
| Trim and fill analysis | 0.63 | 0.01, 1.25 | -2.02, 3.29 | 0.046 | 1.47 [0.56, 3.74] | 90 [77, 96] |


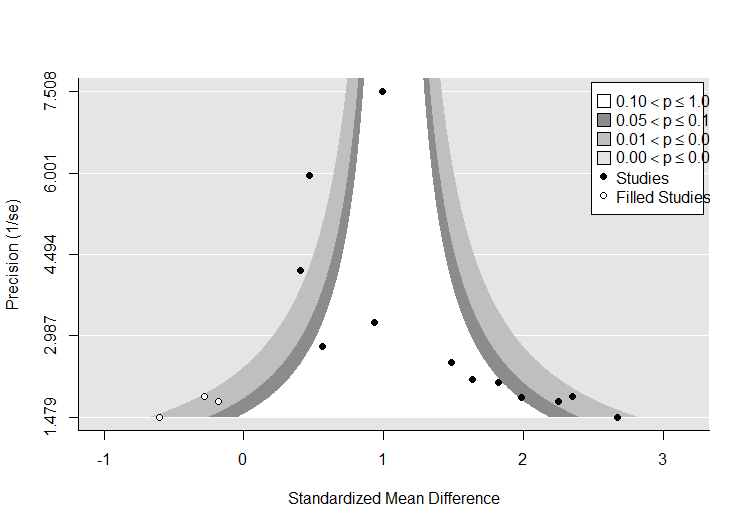

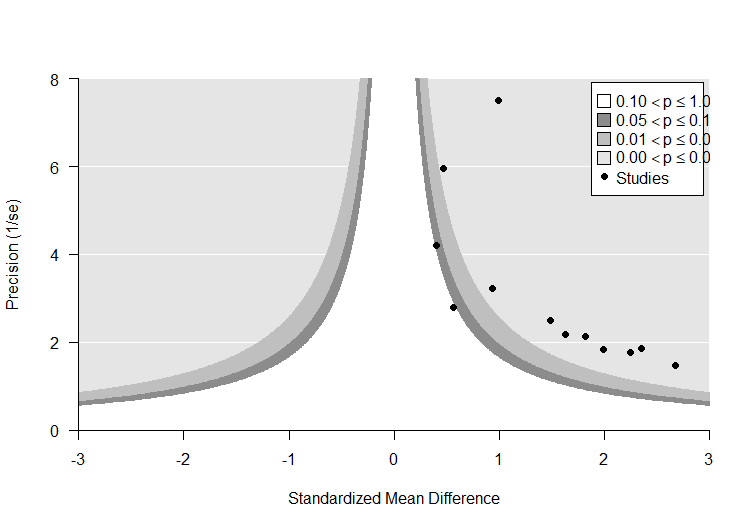
**Insulin**

**Figure S6:** Contour-enhanced funnel plot for studies measuring insulin following trim and fill analysis.

**Figure S5:** Contour-enhanced funnel plot for studies measuring insulin.

| **Model** | **Mean intervention effect** | **95% confidence interval** | **95% prediction interval** | **P-value** | **Tau-squared [95% CI]** | **I-squared [95% CI]** |
| --- | --- | --- | --- | --- | --- | --- |
| Original | 1.33 | 0.88, 1.77 | -0.25, 2.91 | <0.001 | 0.45 [0.13, 1.56] | 83 [58, 95] |
| Trim and fill analysis | 1.07 | 0.57, 1.58 | -0.93, 3.08 | <0.001 | 0.79 [0.26, 2.29] | 88 [71, 96] |

**Supplementary Appendix G:** Sensitivity analyses using different correlation coefficients

**Energy intake**

| **r** | **Mean intervention effect** | **95% confidence interval** | **95% prediction interval** | **P-value** | **Tau-squared [95% CI]** | **I-squared [95% CI]** |
| --- | --- | --- | --- | --- | --- | --- |
| 0.3 | -0.18 | -0.58, 0.22 | -1.56, 1.20 | 0.375 | 0.15 [0.02, 1.66] | 78 [32, 97] |
| 0.5 | -0.19 | -0.59, 0.21 | -1.60, 1.22 | 0.345 | 0.16 [0.03, 1.67] | 81 [41, 98] |
| 0.7 | -0.20 | -0.60, 0.19 | -1.66, 1.25 | 0.314 | 0.17 [0.04, 1.69] | 86 [56, 98] |
| 0.9 | -0.22 | -0.61, 0.18 | -1.73, 1.30 | 0.284 | 0.19 [0.05, 1.71] | 93 [79, 99] |

**Energy expenditure**

| **r** | **Mean intervention effect** | **95% confidence interval** | **95% prediction interval** | **P-value** | **Tau-squared [95% CI]** | **I-squared [95% CI]** |
| --- | --- | --- | --- | --- | --- | --- |
| 0.3 | 0.74 | 0.34, 1.13 | -0.12, 1.59 | <0.001 | 0.03 [0.00, 0.70] | 16 [0, 80] |
| 0.5 | 0.72 | 0.37, 1.08 | -0.12, 1.56 | <0.001 | 0.04 [0.00, 0.74] | 23 [0, 85] |
| 0.7 | 0.70 | 0.39, 1.02 | -0.15, 1.56 | <0.001 | 0.05 [0.00, 0.79] | 36 [0, 91] |
| 0.9 | 0.68 | 0.40, 0.96 | -0.23, 1.59 | <0.001 | 0.06 [0.00, 0.83] | 69 [0, 97] |

**Glucose**

| **r** | **Mean intervention effect** | **95% confidence interval** | **95% prediction interval** | **P-value** | **Tau-squared [95% CI]** | **I-squared [95% CI]** |
| --- | --- | --- | --- | --- | --- | --- |
| 0.3 | 1.08 | 0.57, 1.59 | -0.71, 2.87 | <0.001 | 0.59 [0.10, 2.00] | 76 [34, 91] |
| 0.5 | 1.11 | 0.60, 1.62 | -0.74, 2.97 | <0.001 | 0.64 [0.71, 2.10] | 82 [55, 94] |
| 0.7 | 1.15 | 0.64, 1.66 | -0.78, 3.09 | <0.001 | 0.71 [0.26, 2.21] | 89 [76, 96] |
| 0.9 | 1.21 | 0.69, 1.72 | -0.83, 3.25 | <0.001 | 0.79 [0.36, 2.31] | 97 [93, 99] |

**Insulin**

| **r** | **Mean intervention effect** | **95% confidence interval** | **95% prediction interval** | **P-value** | **Tau-squared [95% CI]** | **I-squared [95% CI]** |
| --- | --- | --- | --- | --- | --- | --- |
| 0.3 | 1.28 | 0.83, 1.73 | -0.24, 2.80 | <0.001 | 0.41 [0.07, 1.48] | 79 [41, 92] |
| 0.5 | 1.33 | 0.88, 1.77 | -0.25, 2.91 | <0.001 | 0.45 [0.13, 1.56] | 83 [58, 95] |
| 0.7 | 1.38 | 0.94, 1.82 | -0.27, 3.03 | <0.001 | 0.50 [0.19, 1.63] | 88 [74, 96] |
| 0.9 | 1.44 | 1.00, 1.88 | -0.31, 3.19 | <0.001 | 0.57 [0.26, 1.72] | 95 [90, 98] |

**Supplementary Appendix H:** Sensitivity analyses excluding studies using doses that were sub-anorectic or prevented hyperglycaemia

**Energy intake**

| **Model** | **Mean intervention effect** | **95% confidence interval** | **95% prediction interval** | **P-value** | **Tau-squared [95% CI]** | **I-squared [95% CI]** |
| --- | --- | --- | --- | --- | --- | --- |
| Original | -0.19 | -0.59, 0.21 | -1.60, 1.22 | 0.345 | 0.16 [0.03, 1.67] | 81 [41, 98] |
| Excluding study ^20^ | -0.24 | -0.72, 0.25 | -2.46, 1.99 | 0.344 | 0.21 [0.04, 3.59] | 86 [52, 99] |

**Glucose**

| **Model** | **Mean intervention effect** | **95% confidence interval** | **95% prediction interval** | **P-value** | **Tau-squared [95% CI]** | **I-squared [95% CI]** |
| --- | --- | --- | --- | --- | --- | --- |
| Original | 1.11 | 0.60, 1.62 | -0.74, 2.97 | <0.001 | 0.64 [0.71, 2.10] | 82 [55, 94] |
| Excluding study ^23^ | 1.22 | 0.72, 1.72 | -0.51, 2.95 | <0.001 | 0.54 [0.09, 1.93] | 78 [38, 93] |

**Supplementary Appendix I:** Leave-one-out meta-analyses

**Energy intake**

| **Study left out** | **Mean intervention effect** | **95% confidence interval** | **P-value** | **Tau-squared** | **I-squared** |
| --- | --- | --- | --- | --- | --- |
| Bagger et al.^19^ | -0.07 | -0.42, 0.27 | 0.685 | 0.09 | 73 |
| Cegla et al.^20^ | -0.24 | -0.72, 0.25 | 0.344 | 0.20 | 86 |
| Geary et al.^21^ | -0.12 | -0.61, 0.37 | 0.636 | 0.19 | 77 |
| Izzi-Engbeaya et al.^22^ | -0.35 | -0.70, 0.01 | 0.054 | 0.08 | 65 |
| Stahel et al.^23^ | -0.21 | -0.71, 0.30 | 0.419 | 0.22 | 86 |

**Energy expenditure**

| **Study left out** | **Mean intervention effect** | **95% confidence interval** | **P-value** | **Tau-squared** | **I-squared** |
| --- | --- | --- | --- | --- | --- |
| Cegla et al.^20^ | 0.66 | 0.25, 1.07 | 0.002 | 0.04 | 24 |
| Chakravarthy et al.^24^ | 0.80 | 0.45, 1.15 | <0.001 | 0.01 | 11 |
| Salem et al.^25^ | 0.68 | 0.32, 1.04 | <0.001 | 0.03 | 20 |
| Stahel et al.^23^ | 0.69 | 0.24, 1.14 | 0.003 | 0.05 | 26 |
| Tan et al.^26^ | 0.78 | 0.36, 1.19 | <0.001 | 0.05 | 25 |

**Glucose**

| **Study left out** | **Mean intervention effect** | **95% confidence interval** | **P-value** | **Tau-squared** | **I-squared** |
| --- | --- | --- | --- | --- | --- |
| Arafat et al.^27^a | 1.20 | 0.67, 1.72 | <0.001 | 0.62 | 81 |
| Arafat et al.^27b^ | 1.16 | 0.61, 1.71 | <0.001 | 0.70 | 83 |
| Bagger et al.^19^ | 1.13 | 0.57, 1.68 | <0.001 | 0.71 | 84 |
| Cegla et al.^20^ | 1.05 | 0.51, 1.59 | <0.001 | 0.66 | 83 |
| Chernish et al.^28a^ | 1.16 | 0.62, 1.71 | <0.001 | 0.69 | 83 |
| Chernish et al.^28b^ | 0.99 | 0.50, 1.48 | <0.001 | 0.53 | 80 |
| Izzi-Engbeaya et al.^22^ | 1.15 | 0.60, 1.71 | <0.001 | 0.70 | 83 |
| Lockton & Poucher^29^ | 1.18 | 0.64, 1.72 | <0.001 | 0.66 | 82 |
| Ranganath et al.^30^ | 1.09 | 0.58, 1.62 | <0.001 | 0.69 | 84 |
| Salem et al.^25^ | 1.06 | 0.54, 1.58 | <0.001 | 0.65 | 84 |
| Schjoldager et al.^31^ | 1.11 | 0.56, 1.66 | <0.001 | 0.71 | 84 |
| Stahel et al.^23^ | 1.22 | 0.72, 1.72 | <0.001 | 0.54 | 78 |
| Tan et al.^26^ | 0.97 | 0.51, 1.44 | <0.001 | 0.47 | 79 |

**Insulin**

| **Study left out** | **Mean intervention effect** | **95% Confidence interval** | **P-value** | **Tau-squared** | **I-squared** |
| --- | --- | --- | --- | --- | --- |
| Arafat et al.^27a^ | 1.33 | 0.84, 1.81 | <0.001 | 0.51 | 85 |
| Arafat et al.^27b^ | 1.31 | 0.83, 1.79 | <0.001 | 0.50 | 85 |
| Bagger et al.^19^ | 1.38 | 0.90, 1.86 | <0.001 | 0.48 | 84 |
| Cegla et al.^20^ | 1.25 | 0.80, 1.69 | <0.001 | 0.42 | 83 |
| Chernish et al.^28a^ | 1.29 | 0.82, 1.77 | <0.001 | 0.49 | 85 |
| Chernish et al.^28b^ | 1.23 | 0.81, 1.66 | <0.001 | 0.38 | 82 |
| Izzi-Engbeaya et al.^22^ | 1.38 | 0.89, 1.86 | <0.001 | 0.49 | 80 |
| Ranganath et al.^30^ | 1.42 | 0.97, 1.87 | <0.001 | 0.41 | 78 |
| Salem et al.^25^ | 1.26 | 0.80, 1.71 | <0.001 | 0.44 | 84 |
| Schjoldager et al.^31^ | 1.40 | 0.94, 1.86 | <0.001 | 0.44 | 83 |
| Stahel et al.^23^ | 1.42 | 0.97, 1.87 | <0.001 | 0.39 | 80 |
| Tan et al.^26^ | 1.28 | 0.81, 1.75 | <0.001 | 0.47 | 85 |

NOTE: 95% confidence intervals for tau-squared and I-squared, as well as 95% prediction intervals, were not reported for leave-one-out meta-analysis as these were not outputted by the *leave1out()* fucntion within the *metafor* package.

**Supplementary Appendix J:** Changes in glucose and insulin concentrations over time following acute glucagon administration

Mean values for glucose and insulin concentrations over time from individual studies are presented in figures S5 and S6, respectively.

Studies that infused glucagon continuously are only presented. This was necessary to understand the impact of consistently elevated glucagon concentrations on glucose and insulin levels, removing the influence of a decline in glucagon concentrations on these outcomes. Studies that administered glucagon as a bolus were therefore not presented.


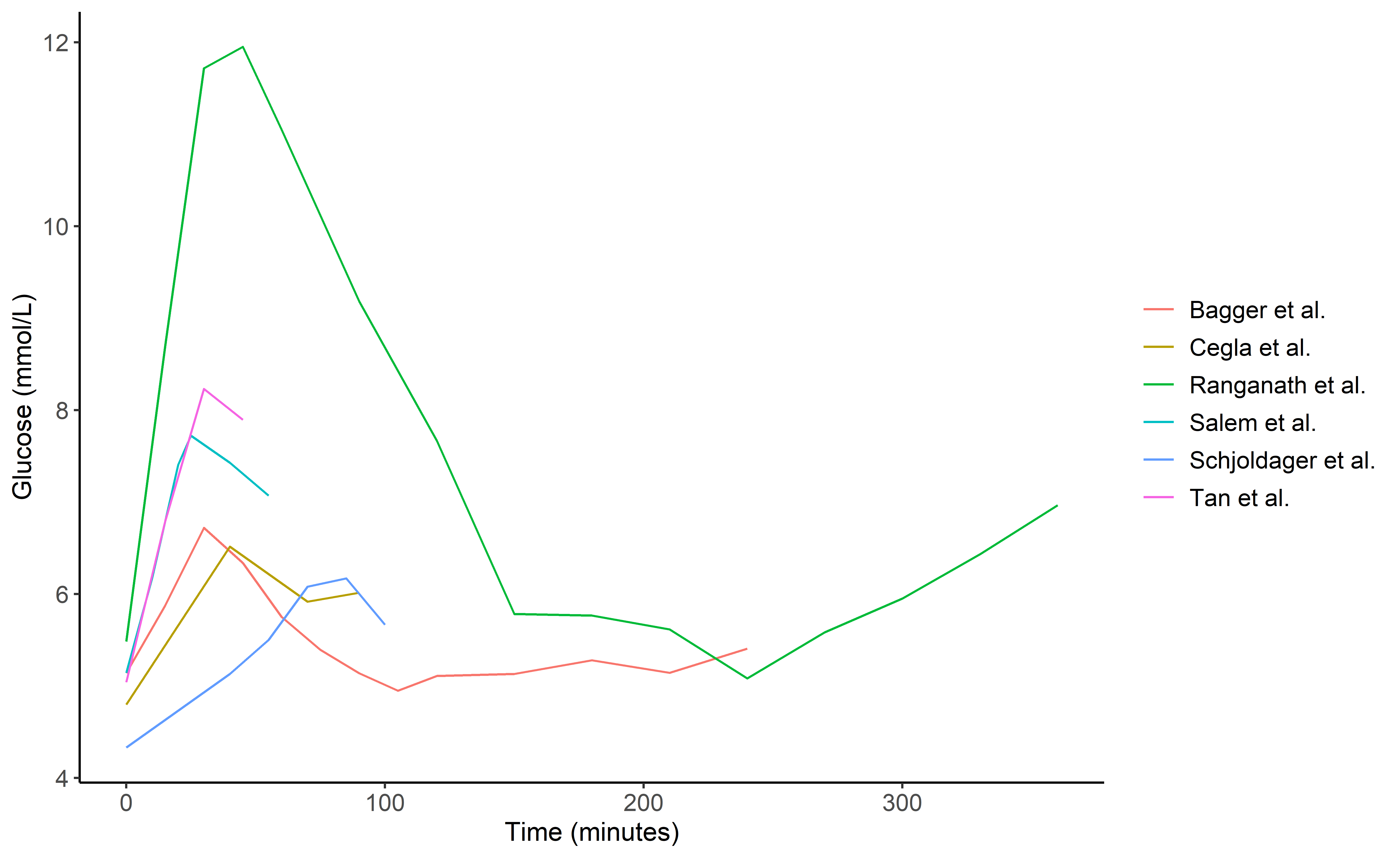
Additionally, only time points for which glucagon was still being infused were included. Again, this was to prevent the confounding of changes in glucose and insulin concentrations by the cessation of glucagon infusion.

**Figure S7:** Changes in mean glucose concentrations over time during continuous glucagon infusion. Individual lines represent values from different studies.


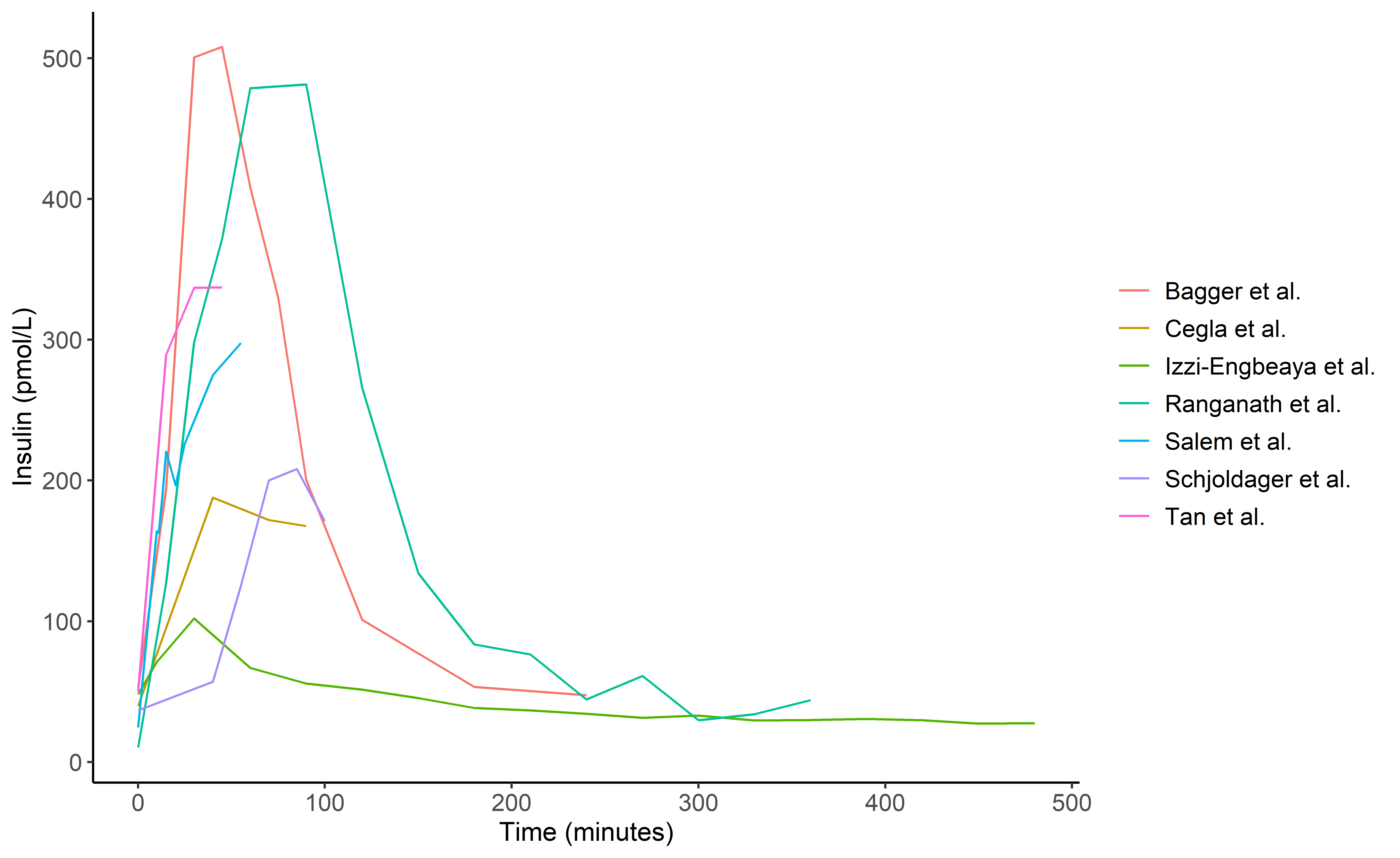


**Figure S8:** Changes in mean insulin concentrations over time during continuous glucagon infusion. Individual lines represent values from different studies.

**REFERENCES**

1 Alford FP, Bloom SR, Nabarro JDN, Hall R, Besser GM, Coy DH *et al.* Glucagon control of fasting glucose in man. *Lancet* 1974; **304**: 974–977.

2 Arvat E, Maccagno B, Ramunni J, Giordano R, DiVito L, Broglio F *et al.* Glucagon is an ACTH secretagogue as effective as hCRH after intramuscolar administration while it is ineffective when given intravenously in normal subjects. *Pituitary* 2000; **3**: 169–73.

3 Breckenridge SM, Raju B, Arbelaez AM, Patterson BW, Cooperberg BA, Cryer PE. Basal insulin, glucagon, and growth hormone replacement. *Am J Physiol Metab* 2007; **293**: E1303–E1310.

4 Broglio F, Gottero C, Prodam F, Destefanis S, Gauna C, Me E *et al.* Ghrelin secretion is inhibited by glucose load and insulin-induced hypoglycaemia but unaffected by glucagon and arginine in humans. *Clin Endocrinol (Oxf)* 2004; **61**: 503–509.

5 Chang HY, Pandolfino JE, Shi G, Boeckxstaens GE, Joehl RJ, Kahrilas PJ. The effect of glucagon-induced gastric relaxation on TLOSR frequency. *Neurogastroenterol Motil* 2003; **15**: 3–8.

6 Cremer GM, Molnar GD, Taylor WF, Moxness KE, Service FJ, Gatewood LC *et al.* Studies of diabetic instability. II. Tests of insulinogenic reserve with infusions of arginine, glucagon, epinephrine, and saline. *Metabolism* 1971; **20**: 1083–1098.

7 Greco A V, Altomonte L, Ghirlanda G, D’Anna LM, Manna R, Caputo S *et al.* Glucagon and glucose tolerance in liver cirrhosis. *Acta Endocrinol (Copenh)* 1988; **118**: 337–345.

8 Kabadi U, Premachandra B. Glucagon administration induces lowering of serum T 3 and rise in reverse T 3 in euthyroid healthy subjects. *Horm Metab Res* 1985; **17**: 667–670.

9 Larsen S, Osnes M, Christensen MS. The effect of glucagon, glucagon-(l-21)-peptide, and placebo on duodenal pressure activity in healthy subjects. *Scand J Gastroenterol* 1986; **21**: 634–640.

10 Liljenquist JE, Rabin D. Lack of a role for glucagon in the disposal of an oral glucose load in normal man. *J Clin Endocrinol Metab* 1979; **49**: 937–9.

11 Massara F, Martelli S, Cagliero E, Camanni F, Molinatti GM. Influence of glucagon on plasma levels of potassium in man. *Diabetologia* 1980; **19**: 414–417.

12 Meier JJ, Ritter PR, Jacob A, Menge BA, Deacon CF, Schmidt WE *et al.* Impact of exogenous hyperglucagonemia on postprandial concentrations of gastric inhibitory polypeptide and glucagon-like peptide-1 in humans. *J Clin Endocrinol Metab* 2010; **95**: 4061–4065.

13 Penick SB, Hinkle LE, Paulsen EG. Depression of food intake induced in healthy subjects by glucagon. *N Engl J Med* 1961; **264**: 893–897.

14 Pontiroli AE, Perfetti MG, Andreotti AC, Fattor B, Monti LD, Pozza G. Metabolic effects of graded glucagon infusions in man: Inhibition of glucagon, insulin, and somatostatin response to arginine. *Metabolism* 1993; **42**: 1242–1248.

15 Schade DS, Eaton RP. Modulation of fatty acid metabolism by glucagon in man: IV. Effects of a physiologic hormone infusion in normal man. *Diabetes* 1976; **25**: 978–983.

16 Schade D, Eaton R. The effect of short term physiological elevations of plasma glucagon concentration on plasma triglyceride concentration in normal and diabetic man. *Horm Metab Res* 1977; **9**: 253–257.

17 Sherwin RS, Fisher M, Hendler R, Felig P. Hyperglucagonemia and blood glucose regulation in normal, obese and diabetic subjects. *N Engl J Med* 1976; **294**: 455–461.

18 Turner DA, Audhya TK, Cramp DG, Holdsworth CD, McIntyre N. Effect of glucagon in intravenous glucose tolerance. *BMJ* 1967; **4**: 145–146.

19 Bagger JI, Holst JJ, Hartmann B, Andersen B, Knop FK, Vilsbøll T. Effect of oxyntomodulin, glucagon, GLP-1, and combined glucagon +GLP-1 infusion on food intake, appetite, and resting energy expenditure. *J Clin Endocrinol Metab* 2015; **100**: 4541–4552.

20 Cegla J, Troke RC, Jones B, Tharakan G, Kenkre J, McCullough KA *et al.* Coinfusion of low-dose GLP-1 and glucagon in man results in a reduction in food Intake. *Diabetes* 2014; **63**: 3711–3720.

21 Geary N, Kissileff HR, Pi-Sunyer FX, Hinton V. Individual, but not simultaneous, glucagon and cholecystokinin infusions inhibit feeding in men. *Am J Physiol Integr Comp Physiol* 1992; **262**: R975–R980.

22 Izzi-Engbeaya C, Jones S, Crustna Y, Machenahalli PC, Papadopoulou D, Modi M *et al.* Acute effects of glucagon on reproductive hormone secretion in healthy men. *J Clin Endocrinol Metab* 2020; **105**: 1899–1905.

23 Stahel P, Lee SJ, Sud SK, Floh A, Dash S. Intranasal glucagon acutely increases energy expenditure without inducing hyperglycaemia in overweight/obese adults. *Diabetes, Obes Metab* 2019; **21**: 1357–1364.

24 Chakravarthy M, Parsons S, Lassman ME, Butterfield K, Lee AYH, Chen Y *et al.* Effects of 13-hour hyperglucagonemia on energy expenditure and hepatic glucose production in humans. *Diabetes* 2017; **66**: 36–44.

25 Salem V, Izzi‐Engbeaya C, Coello C, Thomas DB, Chambers ES, Comninos AN *et al.* Glucagon increases energy expenditure independently of brown adipose tissue activation in humans. *Diabetes, Obes Metab* 2016; **18**: 72–81.

26 Tan TM, Field BCT, McCullough KA, Troke RC, Chambers ES, Salem V *et al.* Coadministration of Glucagon-Like Peptide-1 During glucagon infusion in humans results in increased energy expenditure and amelioration of hyperglycemia. *Diabetes* 2013; **62**: 1131–1138.

27 Arafat AM, Weickert MO, Adamidou A, Otto B, Perschel FH, Spranger J *et al.* The impact of insulin-independent, glucagon-induced suppression of total ghrelin on satiety in obesity and type 1 diabetes mellitus. *J Clin Endocrinol Metab* 2013; **98**: 4133–4142.

28 Chernish SM, Maglinte DDT, Brunelle RL. The laboratory response to glucagon dosages used in gastrointestinal examinations. *Invest Radiol* 1988; **23**: 847–852.

29 Lockton JA, Poucher SM. Single dose glucagon (0.5 mg IV bolus) administration in healthy human volunteers is a robust model for assessment of glycogenolysis. *J Pharmacol Toxicol Methods* 2007; **55**: 86–90.

30 Ranganath L, Schaper F, Gama R, Morgan L, Wright J, Teale D *et al.* Effect of glucagon on carbohydrate-mediated secretion of glucose-dependent insulinotropic polypeptide (GIP) and glucagon-like peptide-1 (7-36 amide) (GLP-1). *Diabetes Metab Res Rev* 1999; **15**: 390–394.

31 Schjoldager B, Lawaetz O, Christiansen J. Effect of pancreatic glucagon and its 1-21 fragment on gastric emptying in man. *Scand J Gastroenterol* 1988; **23**: 726–730.
